# Bioinspired hydrogel-based regenerative engineering strategies for the structural and functional regeneration of skeletal muscles following volumetric muscle loss

**DOI:** 10.1093/rb/rbag175

**Published:** 2026-08-19

**Authors:** Mohammed A Barajaa, Debolina Ghosh, Cato T Laurencin

**Affiliations:** Department of Biomedical Engineering, College of Engineering, Imam Abdulrahman Bin Faisal University, Dammam 34212, Saudi Arabia; The Cato T. Laurencin Institute for Regenerative Engineering, University of Connecticut, Farmington, CT 06030, USA; The Cato T. Laurencin Institute for Regenerative Engineering, University of Connecticut, Farmington, CT 06030, USA; Department of Biomedical Engineering, University of Connecticut, Storrs, CT 06269, USA; Department of Orthopedic Surgery, University of Connecticut Health Center, Farmington, CT 06030, USA; Department of Materials Science & Engineering, University of Connecticut, Storrs, CT 06269, USA; Department of Chemical & Bimolecular Engineering, University of Connecticut, Storrs, CT 06269, USA

**Keywords:** skeletal muscle, volumetric muscle loss, regenerative engineering, muscle regeneration, hydrogels based biofabrication approaches, alignment

## Abstract

Skeletal muscle is a highly organized tissue composed of densely packed, uniaxially aligned myofibers that enable directional force generation and normal motor function. Although skeletal muscle has robust endogenous regenerative capacity, this response is overwhelmed by volumetric muscle loss, which heals through fibrosis and scarring rather than functional regeneration, causing permanent structural and functional deficits. Current treatments, including autologous muscle transfer, remain limited by donor-site morbidity, restricted tissue availability and incomplete restoration of muscle form and function. Thus, a major knowledge gap remains: the lack of an implantable, scalable construct capable of recapitulating native muscle architecture, mechanics and function. Skeletal muscle regenerative engineering (SMRE), which integrates suitable cell sources, advanced biomaterials and biomimetic fabrication strategies, offers a promising approach to address this gap. Hydrogels are particularly attractive for SMRE because of their high water content, muscle-like viscoelasticity, tunable properties and compatibility with biochemical functionalization and diverse fabrication methods. Herein, we critically review hydrogel-based SMRE strategies for engineering myo-mimetic skeletal muscle constructs, including cell sources, hydrogel systems, anisotropic organization cues and fabrication methods. We further examine key translational barriers, including vascularization, immune response, cell maturation, scalability and manufacturing reproducibility, and outline actionable future directions for clinical translation.

## Graphical abstract

**Graphical Abstract:**
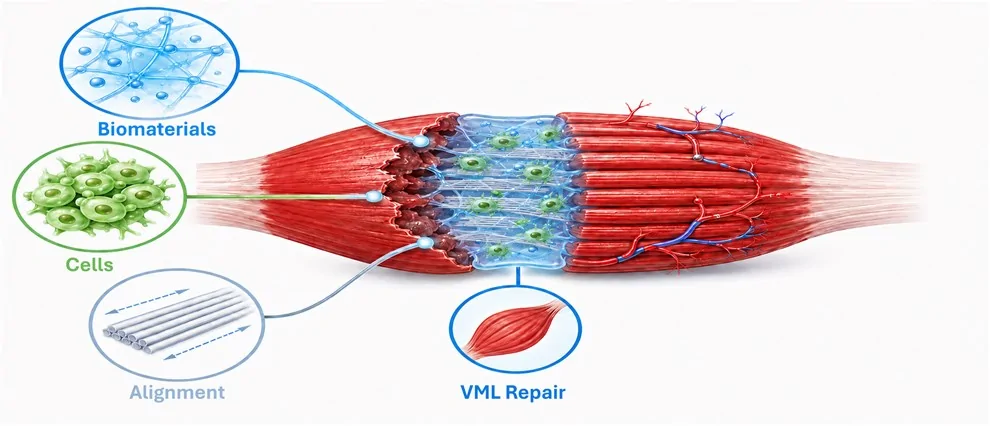

## Introduction

Among all the tissues within the body, the skeletal muscle tissue takes the lead, comprising the highest proportion and making up approximately 40–45% of the total body weight [1]. The primary function of the skeletal muscle is to facilitate body movement through moving bones and other body parts [2, 3]. Uniquely, the function of skeletal muscle is directly correlated to its well-organized microstructure, which is characterized by bundles of densely packed and uniaxially aligned myofibers [4–6]. Myofibers are the largest functional apparatus of the skeletal muscle, which originate during muscle development from the progressive fusion of undifferentiated muscle progenitors called myoblasts into multinucleated and well-elongated myotubes [7]. These myotubes progress into fully mature muscle fibers, each composed of hundreds to thousands of myofibrils [7]. The sarcomere, serving as the fundamental unit of a myofibril, features highly organized myofilaments, such as actin and myosin, crucial for orchestrating muscle contraction and relaxation [8].

Owing to the relatively superficial anatomical location of the skeletal muscle tissue, it is prone to injury. In cases of minor injuries, i.e. muscle loss of less than 20%, the skeletal muscle displays a unique endogenous capacity for regeneration [1]. However, in cases of severe injuries, i.e. muscle loss greater than 20%, the skeletal muscle’s endogenous regenerative capability is impaired, resulting in extensive and irreversible fibrosis, scarring and functional impairments—a condition known as volumetric muscle loss (VML) [9]. Such a condition can result from many events, including but not limited to tumor resections, work/car accidents or compact injuries [10–12]. Regardless of the cause, VML significantly impacts the patients’ movement and can lead to progressive muscle loss and degeneration if left untreated [12–14].

To interrogate VML pathophysiology and to benchmark emerging regenerative therapies, a range of standardized preclinical animal models has been established, most commonly involving surgical excision of a defined volume of muscle (e.g., the tibialis anterior or quadriceps) in rodents, with larger-animal models also employed to better approximate human injury dimensions [15–17]. As the present review focuses on hydrogel-based regenerative engineering strategies rather than on the development of injury models, readers seeking detailed descriptions of VML model design, standardization and functional assessment are directed to these dedicated methodological references [15–17].

### Current treatment strategies and their limitations

Currently, autologous muscle transfer, where muscle tissues with intact blood and nerve supply are harvested from a healthy donor site and grafted into the injury site, is considered to be the gold standard for the treatment of VML [18–20]. This treatment approach can effectively regenerate the lost muscle tissue and restore its impaired function [21, 22]. However, donor site morbidity, increased surgical times and the limited availability of donor tissues, especially in severely injured individuals, are the significant drawbacks of this approach [1].

Another clinical treatment includes physical therapy, which applies specific regimens of exercises to the injured muscle [23]. This treatment approach aims to decrease the progressive degeneration of the muscle tissue by strengthening the remaining muscles and promoting muscle regeneration and healing through stimulating a regenerative microenvironment at the site of injury [23–25]. However, although physical therapy can enhance the repair and recovery of muscle, it often does not lead to substantial structural and functional muscle regeneration within the defect area in VML. Moreover, individuals with excessive VML injuries may not be able to do consistent exercises, thus limiting physical therapy as a viable treatment approach for VML [12].

Acupuncture represents an alternative treatment method for VML, which focuses on electrically stimulating the injured muscle to suppress the expression of myostatin—a growth factor that inhibits muscle growth, leading to muscle cell proliferation and skeletal muscle growth and repair [26, 27]. Similar to physical therapy, acupuncture can improve muscle recovery and functional restoration but cannot facilitate substantial muscle regeneration, especially in severely injured muscles. Furthermore, the optimal electrical stimulation regimens are yet to be identified in this treatment method [12].

### Regenerative engineering

Since current clinical strategies manifest several limitations and cannot achieve satisfactory results for the treatment of VML, a paradigm shift is required to bridge the gap. Regenerative Engineering (RE) is a transdisciplinary approach founded on combining cutting-edge technologies available in disparate fields to develop highly translational technologies for the regeneration of complex tissues and organ systems [28–30]. Within this paradigm, stem cells, advanced biomaterials and imitative fabrication strategies are integral elements for developing *in vitro* bioartificial constructs, which can potentially be used as tissue replacements to foster and accelerate the regenerative process.

For the successful engineering of a skeletal muscle construct (SMC), RE tends to combine the appropriate cell type with a suitable biomaterial that exhibits biomimetic viscoelastic and topographical features. In this frame, various cell types, biomaterials and fabrication strategies have currently been used to produce such constructs. In addition to the skeletal muscle precursors, such as satellite stem cells (SSCs), cells sourced from other tissues, including adipose, bone marrow, umbilical cord, capillaries, blood vessels, skin and pancreas, have been identified as potential candidates for skeletal muscle regenerative engineering (SMRE) [31]. To enhance the therapeutic potential of these cells and their engraftment and localization in the recipient tissue, cells are often incorporated within a biomaterial [32]. Biomaterials such as polyesters [e.g., polylactic acid (PLA), poly(lactic-co-glycolic acid) (PLGA) and polycaprolactone (PCL)] have the capacity to promote muscle cell growth and contribute to skeletal muscle regeneration to a certain extent [33]. However, cytotoxicity concerns mainly related to the acidic degradation byproducts, lack of cell-binding sites and mechanical incompatibility have endorsed the development of alternate biomaterial systems with enhanced cytocompatibility and bio-mimetic properties [34]. Of those, hydrogel-based biomaterials stand out as the most promising candidates for SMRE [4]. Their exceptional characteristics (e.g., higher water content, muscle-like viscoelasticity, tunable rheological properties, the ability to be functionalized with various biochemical cues and the amenability to be easily shaped into different design configurations) have marked them highly desirable for engineering SMCs [32]. In this context, recent advancements in hydrogel-based RE techniques have facilitated the creation of hydrogel-based constructs. These constructs showcase a highly cell-compatible microenvironment with anisotropic cellular organization, contributing to the development of muscle tissues that are both functionally and structurally biomimetic [35, 36].

Herein, a critical and comprehensive overview of the state-of-the-art hydrogel-based RE strategies for engineering SMCs is presented. First, we explore various cell sources that hold potential for SMCs engineering. Subsequently, we describe the primary fabrication strategies employed in SMRE for developing SMCs, with a specific emphasis on hydrogel-based methods. Within this context, we meticulously review the diverse techniques utilized to guide the anisotropic organization of muscle cells within hydrogels.

Furthermore, we provide an extensive review of current hydrogel-based RE methods aimed at fabricating highly myo-mimetic SMCs. This includes detailed insights into techniques such as cast molding, electrospinning, 3D bioprinting, fiber ejection and microfluidic spinning. Finally, we explore future directions pertaining to the scalability of hydrogel-based SMCs, particularly in the context of clinical translation.

## Cell sources for SMRE

The delivery of cells that can contribute to *de novo* muscle regeneration and repair is the basis of cell-based therapies for VML [31, 32]. They have gained much attention over the years due to their effectiveness in treating such trauma [31, 32]. In hydrogel-based RE strategies, the inclusion of cells within the engineered construct during the fabrication process is indispensable for guaranteeing the success of such an approach toward the treatment of excessive muscle injuries [37]. At present, cell-based therapy for SMRE is focused on employing the most suitable cell source in terms of ease of isolation, yield, proliferative and self-renewal capability and, most importantly, myogenic regenerative potential [31, 32]. Recently, the clinical translational potential of cells has also become a primary focus in today’s cell-based research for SMRE [38]. In addition to the skeletal muscle precursors (i.e. SSCs), various cell sources exhibiting myogenic differentiation potential have been identified. These include adipose-derived stem cells (ADSCs), bone marrow-derived mesenchymal stem cells (BMSCs), umbilical cord-derived mesenchymal stem cells (UC-MSCs), perivascular mesenchymal stem cells (PV-MSCs), embryonic stem cells (ESCs) and induced pluripotent stem cells (iPSCs; Figure 1). This section offers an introduction to each of these cell sources, highlights their advantages and disadvantages in the context of VML treatment, and provides insights into their potential clinical utility, as summarized in Table 1.

**Figure 1:**
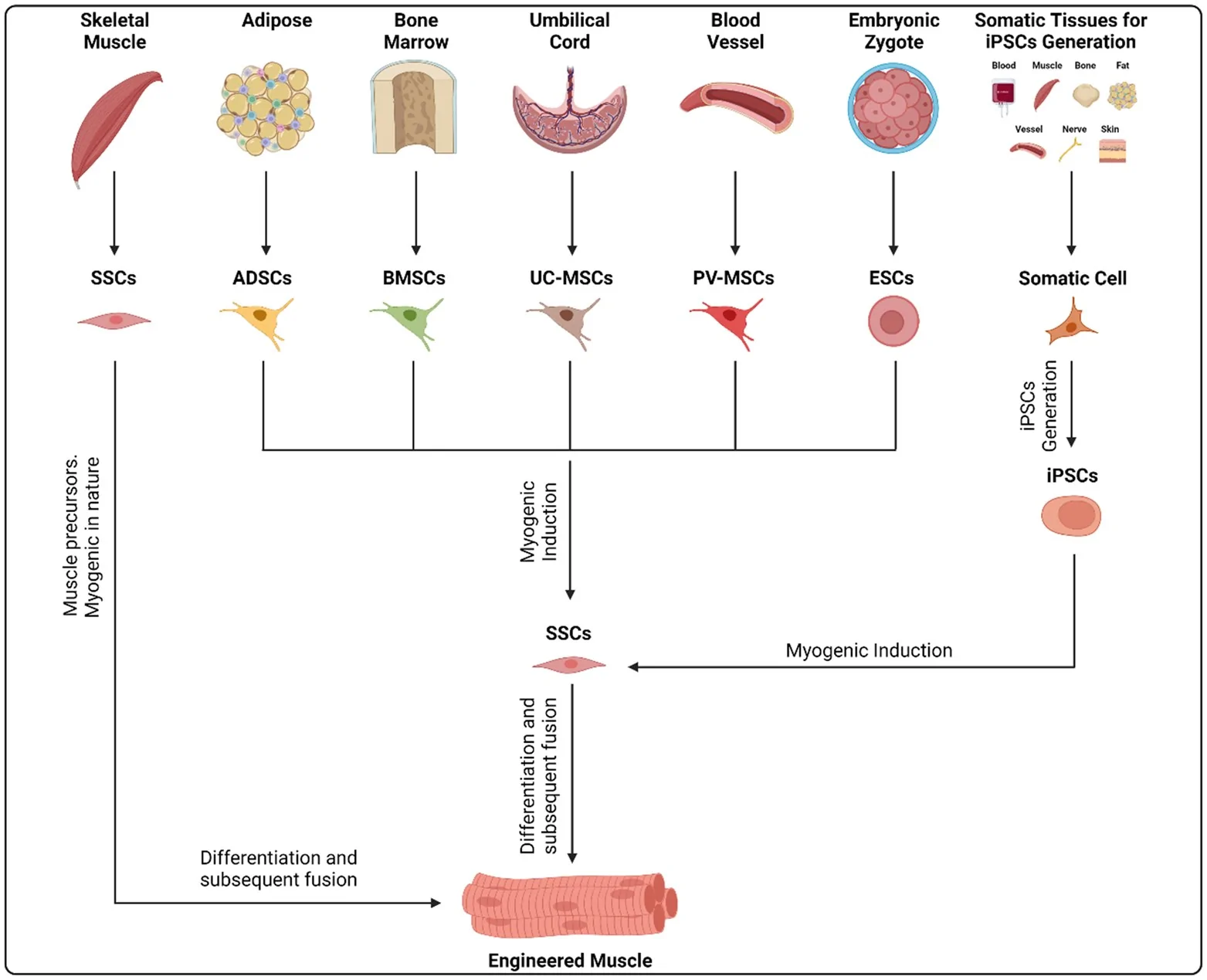
Schematic depicting diverse cell sources recognized for their myogenic potential.

**Table 1:** Comparison of cell sources for myogenic differentiation: clinical translatability, advantages and disadvantages.

Cell type	Ease of isolation	Myogenic induction method	Myogenic potency	Contribution to muscle regeneration	Pros	Cons	Immunogenicity concerns	Clinical translatability	Reference(s)
SSCs	Low	Myogenic in nature, but cultivation in a defined myogenic medium with low serum levels is generally used as a standard method to promote their differentiation into myoblasts and subsequent fusion into myotubes	High	Via fusion into multinucleated myofibers, replenishing the stem cell pool	Native precursors to the skeletal muscle possess remarkable self-renewal and myogenic differentiation capability, absolutely required for skeletal muscle regeneration and maintenance	Isolation requires painful and invasive biopsy procedures, biopsy size is limited due to donor site morbidity, hard to isolate, necessitate further purification steps following isolation, poor cell yield after isolation, lose their stemness, proliferative and regenerative capacity upon repeated passages	Low	Low	[17, 45–48, 50, 51, 53–59, 61–63, 116]
ADSCs	High	Utility of a defined myogenic media supplemented with myogenic growth factors, physically co-culturing ADSCs with myoblasts, applying biophysical stimuli or genetically modifying the ADSCs	Moderate	Via fusion into multinucleated myofibers or immune-privileged behavior	Multipotent and have the potential to differentiate into multiple cell lineages, including the myogenic lineage, isolation is achieved following less invasive and painful procedures, biopsy size is not limited, easy to isolate, high yield after isolation, do not often require post-isolation purification, possess low immunogenicity before differentiation due to the lack of MHC I and II expression	Lose their stemness upon repeated passages, *in vivo* differentiation fate is hard to control, express MHC I and II after differentiation	Low–Moderate	High	[64–69]
BMSCs	Moderate	Culturing in a defined myogenic media, direct co-culturing with myoblasts, biophysical stimuli or genetically modifying the BMSCs to express specific myogenic genes	Low	Via fusion into multinucleated myofibers or immune-privileged behavior	Multipotent and have the potential to differentiate into multiple cell lineages, including the myogenic lineage, biopsy size is not limited, easy to isolate, possess low immunogenicity before differentiation due to the lack of MHC I and II expression	Isolation requires painful and invasive biopsy procedures, poor cell yield after isolation, may require additional purification steps following isolation, lose their stemness upon repeated passages, *in vivo* differentiation fate is hard to control, express MHC I and II after differentiation	Low	Moderate	[70–75, 77]
UC-MSCs	High	Cultivation in myogenic media supplemented with defined concentrations of horse serum, dexamethasone and hydrocortisone for six weeks, or overexpression of myogenic transcription factors via transfection	Low	Via fusion into multinucleated myofibers or immune-privileged behavior	High self-renewal and multi-directional differentiation capability, isolation is painless and comprises no ethical controversy, possess low immunogenicity before differentiation due to the lack of MHC I and II expression and the low HLA-ABC and HLA-DR expression,	Contradicting results regarding their myogenic potential express MHC I and II after differentiation	Low	Moderate	[78–88]
PV-MSCs	Moderate	Cultivation in a defined myogenic media or direct co-culture with myoblasts	Moderate	Via fusion into multinucleated myofibers, replenishing the stem cell pool	Multipotent, can be derived from a variety of tissues, including pancreas, placenta, adipose and skeletal muscle; their isolation can be painless depending on the tissue source	Isolation can be painful and invasive depending on the tissue source, require additional purification steps after isolation, and low cell yield following isolation	Low–Moderate	Moderate	[89–96]
iPSCs	Low–Moderate	Transgenic (overexpression of myogenic transcription factors) and nontransgenic (induction into the myogenic lineage using small molecules or growth factors) based approaches	High	Via fusion into multinucleated myofibers, replenishing the stem cell pool	Can be generated from all somatic cells, can be generated from autologous tissues, unlimited supply, iPSCs generation comprise no ethical controversy, possess an unlimited self-renewal capacity and indefinite proliferative capability, can be induced to become almost any cell type in the body, including muscle cells,	iPSCs generation is generally expensive, hard, requires expert personnel, and is time-consuming; generation efficiency can be low and the generated iPSCs can have some genetic mutations depending on the generation protocol followed	Low	Moderate-high depending on the generation method followed	[97, 99, 102, 106, 107, 109–113, 117]
ESCs	Low	Transgenic (overexpression of myogenic transcription factors) and nontransgenic (induction into the myogenic lineage using small molecules or growth factors) based approaches	High (limited data)	Not well studied	Pluripotent, unlimited self-renewal and proliferative capacity,	Ethical and social controversy, their isolation is technically difficult, can be tumorigenic, differentiation can be difficult to control, risk of rejection and require immunosuppressants for allogenic transplant.	High	Low-not clinically translatable due to ethical and social controversy	[114, 115]

### Satellite stem cells

SSCs are the native precursors to the skeletal muscle tissue residing at the periphery of myofibers and represent <5% of all myonuclei in healthy muscle tissue [39]. They are highly self-renewal, possess a remarkable myogenic differentiation capability and are absolutely essential for skeletal muscle regeneration and maintenance [40]. In response to tissue injury, a substantial reservoir of quiescent (inactive) SSCs becomes activated at the injury site in response to local signals. These activated cells transform into proliferating myoblasts, which, in turn, undergo differentiation and fusion to generate myofibers. This cascade of events contributes significantly to the muscle repair process, highlighting the potent therapeutic potential of these cells in addressing VML injuries [41–43].

SSCs can be obtained from autologous muscles without causing any significant morbidity [44]. Several protocols have been developed for the isolation, purification and expansion of SSCs with successful results [45–48]. After an adequate cell number is obtained via expansion, SSCs can be transplanted back into the injury site to contribute to *de novo* muscle regeneration and improve the functional outcomes [49, 50]. Several studies have tested the therapeutic potential of SSCs toward VML. Transplantation of SSCs in a murine model of VML injury resulted in significant muscle formation and functional recovery two months post-administration compared to the untreated group [51]. Similarly, administrating SSCs in a rat model of VML contributed to *de novo* muscle regeneration and significantly increased the isometric tetanic force production 28 days post-transplantation compared to rats receiving no treatment [52]. Rabbit, dog and pig VML models have also been used as platforms for testing the therapeutic benefits of SSCs toward the treatment of excessive muscle injuries with promising outcomes [17].

Although SSCs are a promising cell source with a well-documented potential for VML treatment, they are not without limitations. For instance, it is hard to isolate a pure population of SSCs. Most isolated cells are often fibroblasts or other non-myogenic cells, necessitating further purification steps via pre-plating, fluorescence-activated cell sorting (FACS) or magnetic-activated cell sorting (MACS) [45, 53, 54]. Yet, these purification steps are complicated, time-consuming and expensive and can significantly affect the overall viability, quality and regenerative potential of the isolated cells [55–57]. In addition, to obtain SSCs, a painful and invasive biopsy would be required [58]. Hence, to avoid prolonged pain and donor site morbidity [59], only a small biopsy can be obtained in clinical settings. Since SSCs represent only 5% of all myonuclei in the skeletal muscle tissue, clinically obtained muscle biopsies can only yield a small number of SSCs that may not be sufficient for clinical utility. While several protocols have been developed for the expansion of SSCs [53, 60–62], it is noteworthy that they often exhibit a reduction in both proliferative capacity and myogenic regenerative potential with repeated passages [63].

Due to the limitations mentioned above, substantial progress has been made over the past decade in exploring alternative cell sources with myogenic potential for VML treatment. These efforts have led to the identification of several other promising cell sources, which are elaborated upon in the following sections.

### Adipose-derived stem cells

ADSCs are mesenchymal stem cells (MSCs) that can be obtained from adipose tissue and possess a multipotent potential. They are adherent, expandable and have the potential to differentiate into multiple cell lineages, including adipogenic, osteogenic, chondrogenic and tenogenic lineages [64]. ADSCs were first identified in adipose tissue in 2001, and since then, adipose tissue has been recognized as a promising cell source for tissue engineering and regenerative medicine [64]. ADSCs are an attractive cell phenotype for RE applications not only for their abundance but also because they do not express major histocompatibility complexes I and II (MHC I and II) [65]. Thus, ADSCs sourced from allogenic donors can be used with minimal immunological responses [65]. Recently, the demonstrated ability of ADSCs to differentiate into the myogenic lineage has positioned them as a potential cell source for the treatment of VML. Several methods have been reported to induce the myogenic differentiation of ADSCs, including the utility of a defined myogenic media supplemented with myogenic growth factors, physically co-culturing ADSCs with myoblasts, and applying biophysical stimuli or genetically modifying the ADSCs [31]. In 2016, Kesireddy [66] demonstrated for the first time the potential of ADSCs for VML treatment. Both SSCs and ADSCs were transplanted into a murine model of VML, revealing similar regeneration capability in terms of fiber formation and cell fusion in the defect site. Compared to SSCs, Kesireddy found that ADSCs contributed significantly to vascularization in the injury site. Although no functional restoration testing was performed, the results suggest that ADSCs could be useful for treating VML.

Several protocols have been established to isolate ADSCs, mainly involving the enzymatic digestion of the adipose tissue for releasing the cells [67]. Unlike a muscle biopsy, an adipose biopsy can be obtained following a minimally invasive procedure that does not cause any donor site morbidity or discomfort to the patient [68]. Moreover, the adipose biopsy size is not limited, and ADSCs isolations are generally efficient, with an average yield of approximately 404K cells per 1 mm^3^ of adipose tissue [69]. Thus, ADSCs hold clear advantages over SSCs concerning safety, expense, biopsy size and cell yield. However, it is noteworthy that ADSCs do express MHC I and II after differentiation, and to date, there has not been a study that has investigated the levels of MHC I and II in ADSCs after myogenic differentiation [65].

### Bone marrow-derived mesenchymal stem cells

Similar to ADSCs, BMSCs represent another adherent and expandable stem cell type that originate from the mesenchymal family and are localized in the bone marrow [70]. They are multipotent and can differentiate into mesodermal tissues, including bone, cartilage, fat, tendon/ligament and skeletal muscles [70]. Due to the similarity between BMSCs and ADSCs in terms of their mesenchymal origin and differentiation potential, multiple comparative studies were carried out to compare their proliferative and myogenic differentiation potential. Although these studies revealed contradicting results regarding their proliferative capacity, the majority have agreed that they both hold the same myogenic differentiation potential [71]. Furthermore, similar to ADSCs, BMSCs originating from allogenic sources could be used with minimal immunological complications due to the absence of MHC I and II expression, marking them highly suitable for clinical transplantation [72].

The isolation of BMSCs has recently been standardized, which often involves obtaining a bone marrow biopsy, followed by filtering the harvested bone marrow to retrieve the cells [73–76]. Although the bone marrow procedure is painful and invasive, it is still advantageous over SSCs biopsy because more tissue can be removed without causing significant functional morbidity [31]. BMSCs myogenic induction protocols follow a similar path to the ADSCs, which primarily involve the culturing in a defined myogenic media, direct co-culturing with myoblasts, biophysical stimuli or genetically modifying the BMSCs to express certain myogenic genes [31].

To date, no published study has directly employed BMSCs for the direct engineering of skeletal muscle. However, multiple studies have utilized BMSCs for the treatment of skeletal muscle injuries, offering valuable insights into the potential behavior of BMSCs in the context of SMRE, particularly for the treatment of VML. For instance, BMSCs isolated from green fluorescent protein (GFP) rats were shown to accelerate the functional recovery one month after administration in a rat model of VML [77]. However, histological tracing analysis revealed that the administered GFP-BMSCs failed to differentiate and fuse into myotubes at the defect site. It was concluded that the enhanced functional recovery observed by the BMSCs administration might have been due to the immunomodulatory properties and anti-inflammatory and paracrine effects of BMSCs, which may have played an indirect role in the healing process, possibly by promoting an appropriate immune environment suitable for tissue regeneration.

### Umbilical cord-derived mesenchymal stem cells

UC-MSCs are mesenchymal stem cells derived from the umbilical cord [78]. They possess a high self-renewal capability and multi-directional differentiation potential [78]. Compared to BMSCs, UC-MSCs can be obtained from umbilical cord tissues discarded after birth, so it is a painless process and comprises no ethical controversy [79]. In addition, they exhibit a higher proliferative capacity than BMSCs with lower immunogenicity due to the reduced human leukocyte antigen (HLA)-DR and HLA-ABC expression and the complete lack of MHC I and II expression, which is useful for clinical transplantation [80].

Several protocols have been developed for isolating UC-MSCs, as comprehensively reviewed by Nagamura-Inoue and He [78], which generally involve enzymatic digestion or explant culture methods [81–83]. The myogenic differentiation of UC-MSCs can be induced following multiple protocols. For instance, cultivating UC-MSCs in a myogenic media supplemented with defined concentrations of horse serum, dexamethasone and hydrocortisone for six weeks induced their differentiation into the myogenic lineage [84]. Furthermore, transfecting UC-MSCs with myoblast determination protein (MyoD) was able to force their induction into the myogenic lineage, resulting in their terminal differentiation and fusion into multinucleated myotubes [85]. These studies suggest that UC-MSCs could be a viable cell source for SMRE.

To date, no published studies have explored the application of UC-MSCs for VML treatment. However, these cells have been under investigation as a potential therapy for muscular dystrophy, although conflicting findings have been reported. For instance, Zucconi *et al.* [86] demonstrated that undifferentiated UC-MSCs administered into murine and dog muscular dystrophy models did not differentiate into skeletal muscle *in vivo*; however, the animals displayed significantly improved functional restoration in comparison to untreated controls. On the contrary, Nunes *et al.* [87] observed some degree of muscle regeneration, wherein a small fraction of mouse UC-MSCs demonstrated the ability to differentiate into myotubes following transplantation into a mouse muscle injury model *in vivo*. While the exact reasoning for these conflicting results is not apparent, a recent mechanistic *in vivo* study suggested that UC-MSCs promote muscle regeneration not by directly fusing into myotubes but by providing a healing cytokine environment due to their immune-privileged behavior [88]. Although these observations indicate that UC-MSCs could be useful for clinical skeletal muscle treatment, more research must be conducted to unravel their true potential in treating excessive muscle injuries, such as in VML.

### Perivascular mesenchymal stem cells

PV-MSCs represent another subset within the mesenchymal family of stem cells, which can be further subcategorized into pericytes and adventitial cells [89]. Adventitial cells are present in the tunica adventitia—the outermost layer of arteries and veins [90, 91]. Although adventitial cells are similar to pericytes in terms of their developmental features and general MSCs properties, they are a phenotypically and genotypically distinct population of cells [90]. To date, the myogenic potential of adventitial cells has not been investigated, whereas pericytes’ myogenic potential has been studied in greater detail.

Pericytes are an MSC population found at different intervals along capillaries and microvessel walls [90]. Recent studies found that pericytes are present in various tissues, including the pancreas, placenta, adipose tissue and skeletal muscle [92]. Given that adipose tissue serves as a source of pericytes, obtaining them in abundance can be achieved with minimal discomfort to the patient. However, regardless of their source, pericytes obtained from all of the tissues mentioned above possess myogenic potential. In addition, it was recently discovered that pericytes contribute to normal skeletal muscle development by differentiating into SSCs and muscle fibers [93]. From these observations, it was concluded that pericytes’ myogenesis is a process that naturally occurs during skeletal muscle development and increases in response to acute muscle injuries.

Pericyte isolation can be achieved through enzymatic digestion or explant culture methods, depending on the tissue of origin [94]. Following isolation, pericytes can further be purified using one of the well-established purification methods, including FACS or MACS. For an in-depth exploration of pericyte isolation and purification methods, readers are encouraged to read the comprehensive review article authored by Bryan and D’Amore [95].

Several methods have been used to induce the myogenic differentiation of pericytes, including cultivation in a myogenic differentiation media or the direct co-culture with myoblasts. The first study that investigated the myogenic potential of pericytes for skeletal muscle therapies was conducted by Dellavalle *et al.* [94]. In this study, pericytes from human skeletal muscle biopsies were obtained and expanded to twenty population doublings. This massive expansion yielded at least one billion cells from a single muscle biopsy, which they considered sufficient for clinical utility. Following expansion, pericytes were induced to differentiate into the myogenic lineage. This was done by cultivating them in a myogenic differentiation medium or directly co-culturing them with myoblasts. Both methods efficiently promoted the differentiation of pericytes into multinucleated myotubes. Compared to other myogenic cells, it was found that the myogenic differentiation potential of pericytes was up to an order of magnitude higher. However, their myogenic differentiation potential was not higher than SSCs. To investigate the *in vivo* myogenic potential of pericytes, Dellavalle *et al.* injected them into a murine muscle injury model. They found that they could successfully engraft at the injection site and significantly contribute to *de novo* muscle regeneration. These findings suggest that pericytes could be highly useful for SMRE, especially since they can easily be obtained from an attractive biopsy site, such as the adipose tissue.

While pericytes hold great myogenic potential, no study to date has employed them for SMRE, particularly in the context of VML. However, Fuoco *et al.* [96] conducted a study that drew guidelines for tissue engineers on the utility of pericytes for SMRE. Here, pericytes derived from pig’s skeletal muscles were induced to differentiate into the myogenic lineage by cultivating them in a myogenic media with a low serum content. It was found that pericytes could spontaneously form myotubes under these culture conditions. However, when pericytes were incorporated within polyethylene glycol (PEG)-fibrinogen hydrogels and cultivated under the same culture conditions, their myogenic differentiation was shown to be significantly enhanced both *in vitro* and *in vivo*. While preliminary, these findings have shed light on the potential utility of pericytes for SMRE. Nevertheless, more research must be conducted to reveal their potential toward VML treatment.

### Induced pluripotent stem cells

Since their first successful generation in 2006 [97], iPSCs have marked a significant landmark in the field of biomedical science [98]. iPSCs are somatic cells that have been reprogrammed to a pluripotent state [99]. A wide variety of somatic cells, including blood, bone, muscle, fat, nerve, endothelial and skin cells, can be used as the starting source for the generation of iPSCs [100–105]. Once in the pluripotent state, they can be induced to become almost any cell phenotype, including SSCs [106]. Compared to primary SSCs, iPSCs possess an unlimited self-renewal capacity and can proliferate indefinitely *in vitro* without losing their pluripotency, creating a theoretically unlimited source of cells [98].

The primary advantage of iPSCs is that they can be generated from autologous tissues, thus avoiding immune rejection following transplantation or incompatibility [107]. However, out of all somatic cells, skin fibroblasts are often used to generate iPSCs due to their abundance and high yield after isolation [31]. In addition, skin fibroblasts can be obtained through a simple skin biopsy, which offers a clear advantage over SSCs biopsy for clinical practices.

Post-biopsy, pluripotency must be induced in the cells. In 2006, Takahashi and Yamanaka [97] determined that four transcription factors must be delivered to the cells’ nuclei to induce their pluripotency. These factors are best known by the term ‘Yamanaka factors’ and are octamer-binding transcription factor-3/4 (Oct3/4), SRY-related high-mobility-group (HMG)-box protein-2 (Sox2), c-Myc and Kruppel-like factor-4 (Klf4) [97]. However, the determining factor for the success of iPSCs generation is the method used for delivering these transcription factors to the cell’s nuclei. Several delivery methods have been proposed to date with pros and cons regarding clinical safety and reprogramming efficiency, including MMLV-derived retrovirus, lentivirus, piggyBac, adenovirus, Sendai virus, plasmid, replicating EBNA1 episome, minicircle, RNA- and protein-based delivery methods [108]. Following iPSCs generation, they must be appropriately induced into a myogenic lineage [109]. In this context, two main methods have been described, namely, transgenic and nontransgenic approaches [109]. Transgenic-based myogenic induction of iPSCs is achieved by overexpressing master myogenic transcription factors such as MyoD1 and paired box 3/7 (Pax 3/7) [109]. In contrast, the nontransgenic-based approaches are done through the direct differentiation of iPSCs with stepwise induction using small molecules and growth/differentiation factors [109]. These two approaches produce myogenic precursors that can ultimately fuse into myotubes [102, 109–111]. However, because of the absence of gene insertion, the iPSCs differentiated via the direct myogenic induction approach have been shown to display more genetic stability for up to four months *in vitro*, making them more clinically relevant [109, 112].

Due to the relatively short period since the discovery of iPSCs, only a few studies have thus far investigated their potential toward VML treatment. To name a few, Wu *et al.* investigated the therapeutic potential of human iPSCs (hiPSCs) in a murine model of VML [113]. After inducing the differentiation of the hiPSCs toward the myogenic lineage, they were administered into a 30–40% muscle defect. Histological evaluations of the treated muscle revealed abundant engraftment of transplanted cells and *de novo* myofiber formation at the transplantation site with positive expression of acetylcholine receptors (AChRs) and neuromuscular junction (NMJ), indicating their integration with the host’s neural system. Furthermore, cells positive for human paired box protein-7 (Pax-7) were present at the transplantation site, suggesting that the transplanted cells have contributed to replenishing the SSC pool. Finally, *in situ* functional system assessment demonstrated remarkable enhancement in the contractile function in the muscles receiving the hiPSCs treatment compared to the untreated control. While these results provide key evidence for the therapeutic potential of iPSCs in VML injuries, more studies must be conducted aiming to integrate this powerful class of cells with currently existing SMRE-based therapy toward functional and structural muscle regeneration following VML.

### Embryonic stem cells

ESCs are pluripotent stem cells that can be obtained from the inner cell mass of the five- to seven-day-old blastocyte [114]. They possess an unlimited self-renewal and indefinite proliferative capacity. Given that they are pluripotent, they can differentiate into virtually all tissue types [114]. However, compared to all of the above-discussed stem cells, ESCs exhibit significant drawbacks that hamper their clinical utility. For instance, in addition to being ethically and socially controversial due to the need for destructing the human embryo to obtain them, their isolation involves many technical difficulties and often requires highly expert personnel [114]. Furthermore, ESCs are tumorigenic and their differentiation can be difficult to control. Although the myogenic differentiation potential of ESCs has been demonstrated [115], they have not been studied to a meaningful degree, particularly for muscular therapies, due to the above ethical and technical limitations.

## Biomaterials systems for efficient cell delivery toward an effective VML treatment

Indeed, identifying the proper cell source is crucial to ensure a successful cell-based therapy, particularly for the treatment of VML [32]. However, distinguishing the correct cell delivery system might be as vital to successful cell therapy for skeletal muscle injuries [32]. For a cell-based therapy to be successful, it is crucial to ensure high engraftment and maintenance over the viability of the transplanted cells [32]. For SMRE in particular, controlling the spatial organization of the transplanted cells is as crucial for successful structural and functional muscle regeneration following excessive injuries [37]. Although the direct administration of myogenic cells *via* bolus injections has been shown to improve muscle regeneration and functional restoration in some cases [38, 118–120], many other studies have reported contradictory results [32]. The majority of the previous investigations have agreed that, irrespective of their source of origin, injecting myogenic cells *via* bolus injection can significantly impede their therapeutic effects for VML [32]. This is attributed to the fact that cell administration *via* bolus injection often results in poor local retention and engraftment at the injection site [32]. Additionally, myogenic cells possess low viability and survival rates upon transplantation *via* bolus injection *in vivo* [32]. Furthermore, it has been shown that the direct administration of cells *via* bolus injections fails to restore the structural organization of the skeletal muscle, particularly following VML injuries, resulting in suboptimal functional outcomes [121].

To enhance the therapeutic potential of myogenic cells and their engraftment and localization in the recipient tissue, they have been chiefly incorporated within biomaterials, which act as cell delivery vehicles that ensure the safe delivery of the cells into their targeted sites [122]. A key advantage of biomaterials is that they can provide a protective milieu for the transplanted cells, thus prolonging their survival and facilitating better therapeutic outcomes [32]. For example, incorporating the cells in a protective milieu, such as within biomaterials, can avoid many inflammation-related boundaries and considerably enhance efficiency while reducing the number of cells needed for therapy [32, 122]. Additionally, biomaterials can easily be processed to exhibit biomimetic topographical features that can guide the spatial organization of the incorporated cells, which is crucial for immediate structural and functional skeletal muscle repair. Generally, biomaterials in SMRE can be used to deliver undifferentiated myogenic cells or mature myofibers. However, in comparison to delivering undifferentiated myogenic cells, inducing their differentiation within the biomaterial into aligned, mature and contracting myofibers *ex vivo*, followed by their transplantation into the injury site, is more effective in providing an immediate structural repair, prolonging the survival of the transplanted cells and facilitating better *in vivo* functional integration [32]. Furthermore, this approach offers various additional advantages. For instance, it gives the chance to *ex vivo* pre-condition the engineered construct, which allows the incorporated cells to acclimate the biomaterial’s microenvironment [32]. Thus, providing better sustainability against adverse immune responses upon transplantation. An additional yet crucial advantage lies in the ability to effectively prevent mechanical collapse by utilizing a tissue construct with well-defined mechanical properties before transplantation [32]. Furthermore, biomaterials can be designed to meet the patient-specific defect architectures with the so-called personalized medicine, thereby ensuring an immediate return to function [32]. In this context, substantial efforts have been dedicated over the past two decades to developing biomaterial systems for engineering mature and implantable myofibers with myo-mimetic structural organization, aimed at facilitating functional and histological muscle regeneration following VML. These efforts have led to the emergence of three-dimensional (3D) scaffold and hydrogel systems [123], as summarized in Table 2.

**Table 2:** Current biomaterial systems employed in SMRE.

Biomaterial system	Pros	Cons	Reference(s)
3D scaffolds	Can be fabricated using natural, synthetic and composite materials. Can be fabricated with muscle-like architectures. Can be tailored to match the defect geometries. Can support muscle cell growth, structural organization and differentiation. Mechanically competent.	Mostly stiff, which can inhibit muscle cells’ maturation and force production. They are mostly made from synthetic materials, which can result in suboptimal muscle cell growth and maturation. Require the utility of toxic solvents during processing.	[1, 2, 126–134]
Hydrogels	Can be fabricated using natural, synthetic and composite materials. Can be fabricated with muscle-like architectures. Can be tailored to match the defect geometries. Can support muscle cell growth and structural organization. Much softer in comparison to scaffold systems, which can more efficiently regulate muscle cell behavior and promote their myogenic differentiation. Exhibit muscle-like viscoelasticity, which can allow for sufficient force production and contraction by muscle cells.	Mechanically weaker in comparison to scaffold systems.	[32, 135, 137, 144–146]

### Three-dimensional scaffold systems

Scaffolds are porous 3D structures that are often tailored to the size and geometry of the defect and are designed to provide structural niches to facilitate cell adhesion, growth and differentiation [124]. Scaffolds for SMRE can be made using natural or synthetic biomaterials [125]. However, due to the technical difficulty in processing natural materials for scaffold synthesis, synthetic materials such as polylactic acid (PLA), poly(lactic-co-glycolic acid) (PLGA) and polycaprolactone (PCL) are often used. The fabrication process of scaffolds for skeletal muscle tissue regeneration often emphasizes incorporating structural cues within the scaffold’s bulk. This is crucial for guiding the alignment of myogenic cells post-seeding, a key factor in the organized and enhanced formation and maturation of myofibers [1,2]. Various scaffold types including sponges [126], electrospun [127], braided [128], knitted [129], woven [130] and tubular [131] matrices, have been explored and employed to encourage the formation and maturation of myofibers. However, despite the great potential of these scaffolds to promote organized muscle growth both *in vitro* and *in vivo*, they are mostly stiff, which can substantially inhibit the myogenic differentiation of myogenic cells [132, 133], thus preventing sufficient force production and contraction by the muscle tissue after implantation *in vivo* [134]. Moreover, researchers are still seeking an ideal biodegradable scaffold material that can effectively enhance muscle cells’ adhesion, proliferation, differentiation and extracellular matrix (ECM) secretion. This material should also possess suitable viscoelastic properties to enable sufficient force production and contraction by the muscle tissue [12].

### Hydrogel systems

Hydrogels, on the other hand, represent a softer malleable environment that can more efficiently regulate muscle cells’ behavior, promote their myogenic differentiation and allow for sufficient force production and contraction by the muscle cells [32]. Hydrogels are porous 3D polymeric networks with high water content (>90%) and viscoelastic properties matching those observed in the native muscle tissue; thus, they are more favored by muscle cells [32].

Hydrogels can be classified into natural, synthetic or composite (a mixture of synthetic and natural hydrogels), each with its own advantages and disadvantages [135]. For an in-depth discussion on the various classes of hydrogels in SMRE, readers are referred to our published review paper, which provides a comprehensive exploration of this topic [136]. In brief, naturally derived hydrogels are by far the most popular choice for SMRE due to their relatively similar composition to the native ECM [137]. Collagen, fibrin, alginate, gelatin, agarose and chitosan are among the most extensively investigated natural hydrogels for SMRE [32, 135, 137]. Generally, cells can attach and interact better within natural hydrogels due to the presence of cell-adhesion sites within their matrix. However, they possess poor mechanical and biodegradation properties, which can hamper their utility in certain applications [138]. On the other hand, synthetic hydrogels such as poly(ethylene glycol) diacrylate (PEGDA), N-isopropyl acrylamide (NIPAAm), acrylic acid (AA), polyacrylamide (PAAm), polyvinyl alcohol (PVA), polylactic acid (PLA), polyethylene glycol (PEG) and polyurethane (PU) can be produced with controllable mechanical and biodegradation characteristics [116, 139–143]. Yet, synthetic hydrogels lack the ability to promote cell functions due to the absence of cell-adhesion sites crucial for cell attachment and have nonspecific interactions with cells [32, 144]. To overcome the limitations of both natural and synthetic hydrogels, research efforts over the years have led to the emergence of the so-called composite hydrogels. Composite hydrogels are at least one natural and one synthetic material mixed to make up the hydrogel. By mixing natural and synthetic hydrogels, the best attributes of both could be obtained in one system, resulting in an enhanced microenvironmental niche with tunable properties compared to using either alone [145].

In addition to the previously mentioned classes of hydrogels, those derived from the ECM of native decellularized biological tissues have garnered significant attention in SMRE. This is attributed to their outstanding interactions with the *in vivo* milieu, biocompatibility and soft and elastic consistency [146]. Generally, decellularized ECM (dECM)-derived hydrogels are superior to natural, synthetic and composite hydrogels because they contain the original ECM blueprint and mimic the structural and mechanical complexity of the native tissues, providing an optimal niche with complex environmental cues for enhanced cell adhesion, growth, differentiation and tissue formation and maturation [146, 147].

Irrespective of their origin, the distinctive characteristics of hydrogel systems make them ideal for applications in SMRE. This is substantiated by the abundance of papers published within the field of SMRE that utilize hydrogels over other biomaterial systems (Figure 2). Therefore, this review article aims to comprehensively review the current advances in hydrogel-based biofabrication methods for engineering SMCs with structural and functional biomimicry, specifically tailored for integrative VML treatment.

**Figure 2:**
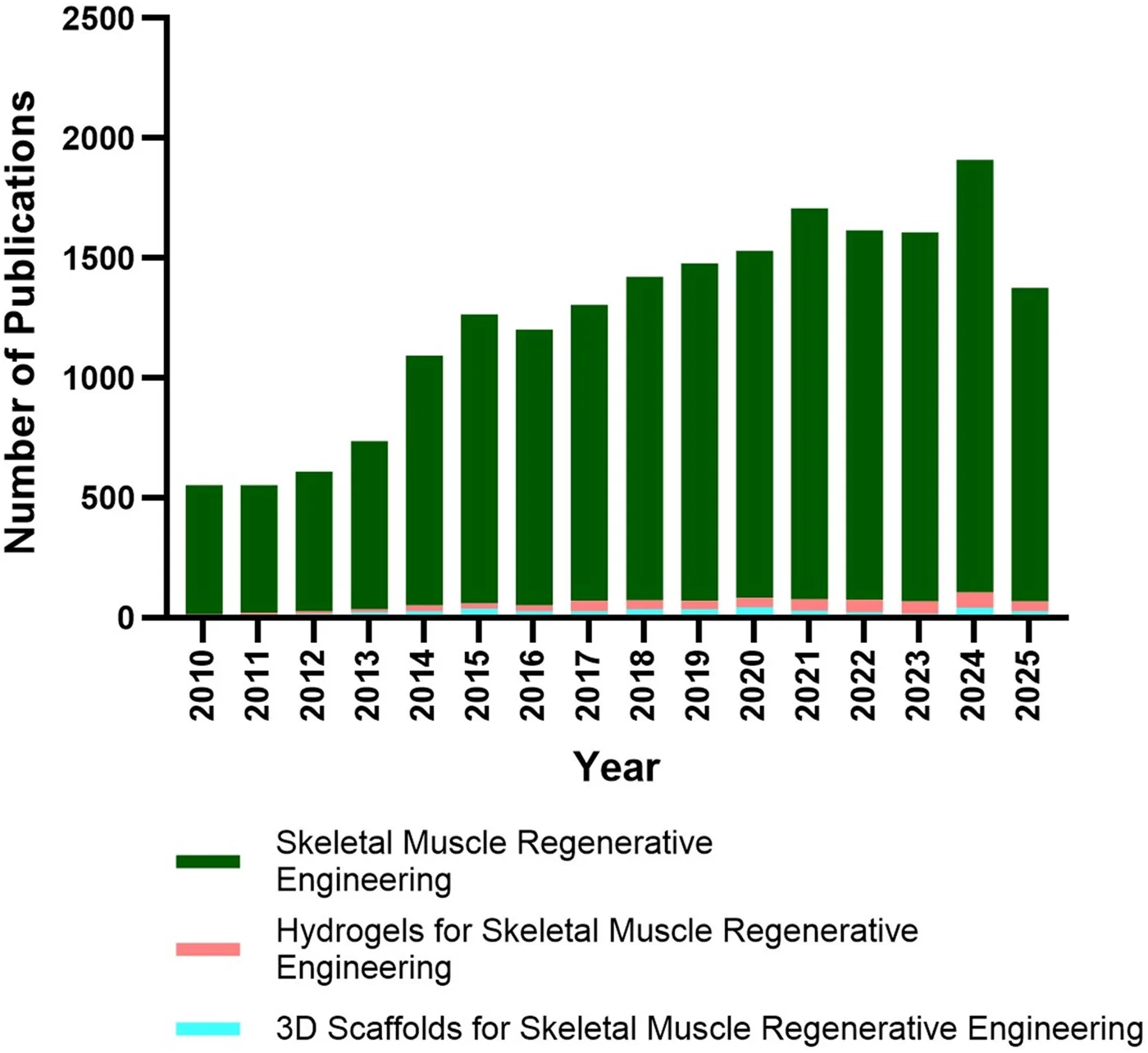
Publication trend in the field of skeletal muscle regenerative engineering. (Data obtained from PubMed; search strings: ‘3D scaffolds’ or ‘hydrogels’ and ‘for skeletal muscle engineering’.)

In this context, the next section of this article will start by discussing the different techniques employed to guide the anisotropic organization and alignment of muscle cells within hydrogel systems. Next, a comprehensive overview of the current hydrogel-based RE methods to fabricate SMCs, such as cast molding, electrospinning, 3D bioprinting, fiber ejection and microfluidic spinning, will be included. Finally, future directions concerning the scalability of hydrogel-based SMCs toward clinical translation will be elaborated.

## Strategies for guiding anisotropic cell organization within hydrogel systems

Despite the exceptional characteristics of hydrogels, a current obstacle hindering their direct use in SMRE is the tendency to form a randomly oriented fibril network upon gelation. Given the anisotropic structure of skeletal muscle tissue, which is characterized by bundles of densely packed and uniaxially aligned myofibers, this random orientation of the fibril network within hydrogels can lead to poor and disorganized muscle cell fusion and muscle regeneration [32]. Consequently, this results in a significant reduction in the uniaxial transmission of the produced forces and, thereby, contributes to poor functional outcomes [32, 127].

To overcome this limitation, several RE strategies have been developed and employed to control muscle cell alignment within hydrogels for producing engineered SMCs with a high cellular organization (Figure 3). These strategies can broadly be categorized into (1) exogenous and (2) endogenous cues, each with its own set of advantages and disadvantages, as summarized in Table 3.

**Figure 3:**
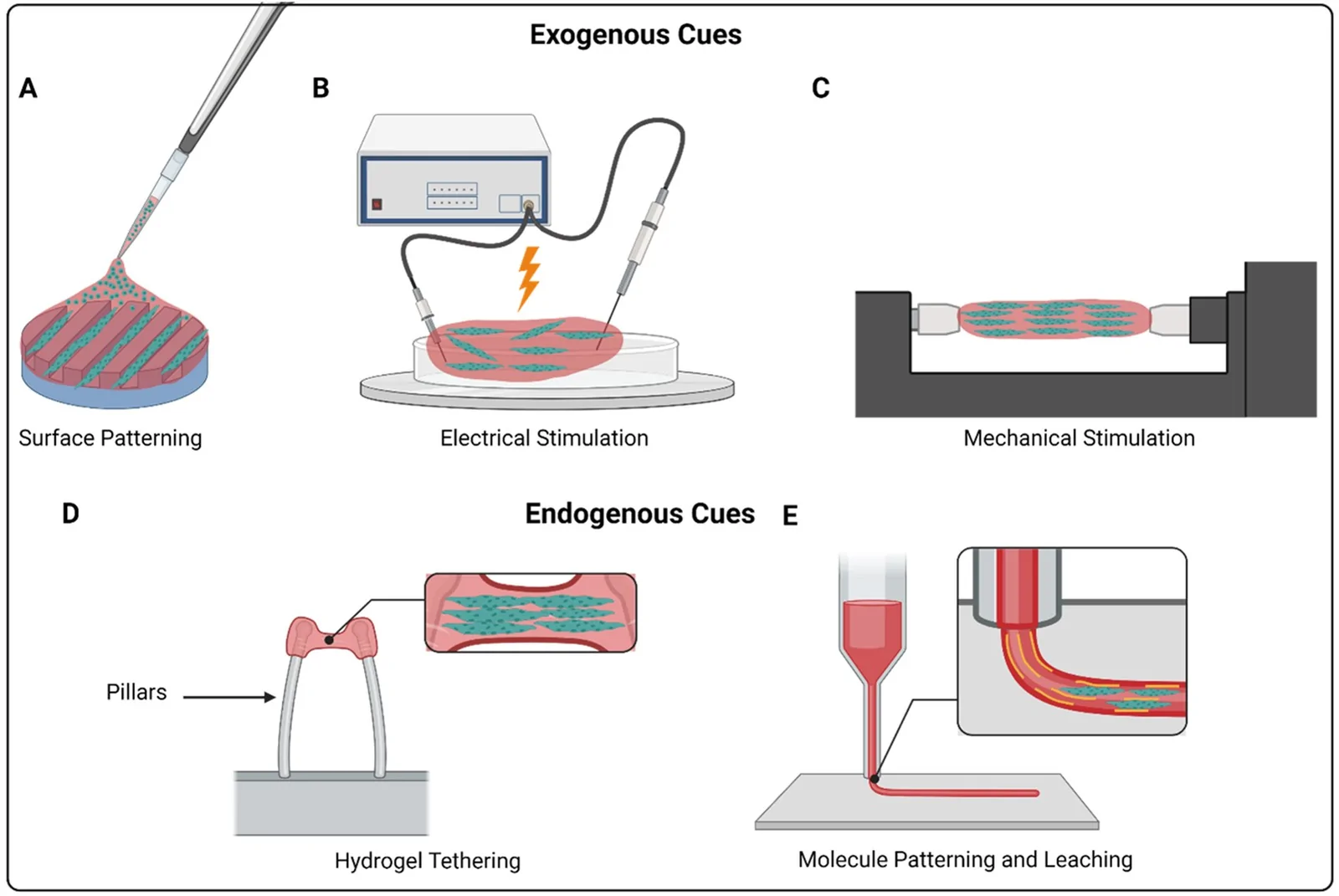
Schematic representation of various strategies employed to guide the anisotropic organization of muscle cells within hydrogel systems. (**A**) Surface patterning, (**B**) electrical stimulation, and (**C**) mechanical stimulation represent exogenous cues used to promote cellular alignment and organization. (**D**) Hydrogel tethering and (**E**) molecule patterning and leaching represent endogenous cues that provide structural guidance within the hydrogel microenvironment.

**Table 3:** Common strategies for guiding the anisotropic organization of muscle cells within hydrogel systems, along with the advantages and disadvantages of each strategy.

Strategy		Pros	Cons	Reference(s)
Exogenous cues	Surface patterning	Simple Efficiently directs muscle cell alignment	Cells need to be in close proximity to the patterned surface to self-align. Unable to align cells in thick or multi-layered constructs	[148–157]
	Electrical stimulation	Can align cells in thick or multi-layered constructs Besides alignment, electrical stimulation can improve muscle cell maturation	Complicated Can affect cell viability Direction of alignment cannot precisely be controlled	[158–162]
	Mechanical stimulation	Simple Efficiently directs muscle cell alignment Can align cells in thick or multi-layered constructs Besides alignment, mechanical stimulation can improve muscle cell maturation	Can affect cell viability at certain strain values Can cause mechanical deterioration of the hydrogel-based construct upon prolonged exposure times to mechanical stretching	[163–165]
endogenous cues	Hydrogel tethering	Simple Efficiently directs muscle cell alignment. Can align cells in thick or multi-layered constructs Alignment is achieved faster than in other approaches Maintains the established alignment during the entire culture time Besides alignment, hydrogel tethering maintains the structural integrity and pre-established pore volume of hydrogel-based constructs during the whole culture time due to its ability to restrict hydrogel cell-mediated contraction	Tethering the hydrogel-based constructs to a pillar system requires skills	[166–176, 178]
	Molecule patterning and leaching	Can align cells in thick or multi-layered constructs Enables guiding the alignment of cells in multiple directions within the same construct	Alignment achieved is not sufficient Only compatible with 3D bioprinting technology Requires an additional step to initiate cell alignment (leaching out the embedded molecules) A large number of cells can be lost during the leaching process	[179–181]

### Exogenous cues for guiding anisotropic cell organization within hydrogel systems

Exogenous cues are those externally applied to cell-landed hydrogels to induce the alignment of the encapsulated cells toward a single uniaxial direction. Surface patterning and electrical and mechanical stimulation are among the most widely used exogenous cues for guiding muscle cell alignment within hydrogel systems.

#### Surface patterning

Surface patterning relies on casting a cell-ladened hydrogel on a pre-fabricated micro-grooved surface obtained through micro-molding processing methods [148–150]. Typically, the microgrooves are confined in a packed and linear fashion for directing muscle cell alignment. Once the cell-laden hydrogel is cast onto the micro-grooved surface, the cells can sense the patterns provided within the underlying substrate and reorient themselves accordingly via a contraction-mediated morphogenetic process guided by boundary constraints (Figure 3A). Thus, the geometric confinement encourages myoblasts to align along the microgroove direction, ultimately resulting in the formation of muscle-like structures [151–153]. One of the earliest attempts that employed the micro-molding technique to produce micro-grooved surfaces for aligning muscle cells within hydrogels was carried out by Cappello *et al.* [154]. Here, C2C12 myoblasts seeded on a gelatin-based micro-grooved surface could self-align along the longitudinal direction of the micro-grooves as early as the first week of culture and form highly aligned myotubes after three weeks of culture. Not only can micro-grooved surfaces be used as platforms for guiding muscle cell alignment, but they can also be used to study the effects of the micro-groove dimensionality, such as width and depth, on the behavior of cell alignment. For instance, Aubin *et al.* [155] developed gelatin methacrylate (GelMA)-based micro-grooved substrates with different groove widths of 50-, 100- and 200-µm. It was found that as the micro-groove width was decreased, cell alignment would significantly increase. The same findings were observed in a different study by Hosseini *et al.* [156], where the alignment of C2C12 cells was shown to be enhanced as the micro-groove width was decreased from a 100- to 50-µm post-seeding on GelMA micro-grooved surfaces. In an attempt to investigate the effect of the micro-groove depth on the behavior of cell alignment, Hume *et al.* [157] created PEG-based micropatterned substrates with different groove depths of 100- and 200- µm. It was found that deeper grooves promoted enhanced cell proliferation compared to wider ones, which in turn provided an improved microenvironment for directing robust cell alignment. These findings suggest that surface patterning can be used as a tool for achieving muscle-like cell organization within hydrogels and that the pattern dimensions, such as micro-groove width and depth, are important parameters to consider when following this approach. However, while surface patterning could successfully direct muscle cell alignment within hydrogels and has unraveled a myriad of scientific questions in this context, it can only do so when the cells are in direct contact with the patterned surface. This indicates that if the cells are further away from the patterned surface, they are more likely to display a disorganized orientation within the hydrogel, thus limiting the utility of this approach to guide cell alignment within thicker hydrogels.

#### Electrical stimulation

Electrical stimulation provides an alternative approach to controlling the organization of muscle cells within hydrogels, facilitating the engineering of hydrogel-based SMCs with a high degree of cellular alignment [158]. Such an approach relies on exposing a cell-ladened hydrogel to an electrical current, which acts as an external cue that can modulate muscle cell behavior, such as directing their alignment (Figure 3B). Since muscle cells are electroactive, they can sense the applied electrical current and act accordingly [159]. It was initially believed that electrical current could only be used as a tool to promote the differentiation of muscle cells toward a mature phenotype [160]. However, recent investigations observed that when muscle cells are exposed to an electrical current, they tend to align themselves parallel to the direction of the applied electrical field [158]. Although micro-grooved hydrogels were used to provide the topographical cue for muscle cell alignment, the cells could not recognize this cue upon excessive growth on the micro-grooved hydrogel [156, 161]. However, when the micro-grooved hydrogel was exposed to an electrical current, the cells deviated from the direction of the hydrogel’s micropatterns and could re-align and follow the direction of the micro-grooves [156, 161].

A key advantage of electrical stimulation is that the applied electrical current can propagate through thick hydrogels. Muscle cells in deeper hydrogel regions can sense the applied current, aligning themselves accordingly, thanks to the intrinsic electrophysiological conductivity of muscle cells. C2C12 cells exposed to an electrical field were able to self-align parallel to the direction of the applied electrical field and showed a similar alignment ratio both in 2D and when encapsulated within thick collagen hydrogels [162]. Therefore, electrical stimulation has the advantage of guiding muscle cell alignment within thick hydrogels, unlike the surface patterning approach. However, it is essential to note that a precise control over the direction of cell alignment can be difficult using this approach. In addition, this approach has yet to identify the optimal electrical stimulation regimens that would induce cell alignment and maturation without affecting their viability.

#### Mechanical stimulation

Mechanical stimulation is a much more effective method for controlling muscle cell alignment within hydrogels than the aforementioned approaches. Mechanical stimulation relies on exposing cell-laden hydrogels to a linear stretching, which can be sensed by muscle cells, directing their alignment and elongation toward the uniaxial direction of stretching (Figure 3C). Several studies have emphasized the effectiveness of mechanical stretching in guiding muscle cell alignment within hydrogels. Chen *et al.* [163] fabricated GelMA-based cell-laden fibers, after which they were subjected to static uniaxial stretching using a pillar well array-based stretching device embedded in a culture system. Here, the cell-laden fibers were uniaxially stretched with various strain ratios of 25, 35 and 45%. Despite the increased cell death observed at increasing strain ratios, C2C12 showed significantly improved spreading, elongation and alignment under uniaxial stretching compared to the C2C12 embedded within fibers receiving no uniaxial stretching. Uniaxial stretching of cell-laden hydrogels can also be applied in a stepwise fashion to prevent cell membrane damage, wherein the hydrogel is gradually stretched over time with a constant rate until the desired stretching length is reached. Pham-Nguyen *et al.* [164] showed that applying stepwise stretching on gelatin-based constructs could significantly improve C2C12 elongation and alignment while maintaining high cell viability. In addition to the stepwise approach, cyclic stretching, which applies a constant linear stretching at different frequencies and strain values, was investigated as a mean to induce muscle cell alignment within hydrogels. This approach tends to mimic the dynamic *in vivo* stretching microenvironment, which in turn stimulates not only their alignment but also helps promote their maturation. Bansai *et al.* [165] developed a custom-made culture device and employed it to apply cyclic stretching on hydrogel fibers ladened with C2C12. The cell-laden hydrogel fibers were subjected to 3% tensile strain at 1 Hz, which resulted in a significantly higher cell alignment and mature myotube formation compared to the cultured monolayer and the nonstretched cell-laden controls. Although 70% of myoblasts successfully fused into mature and highly aligned myotubes when subjected to cyclic stretching, it was observed that cyclic stretching decreased cell viability.

Indeed, mechanical stimulation offers a great tool to guide robust cell alignment within hydrogels and help promote their maturation. However, concerns regarding cell viability and mechanical deterioration of the hydrogel-based construct upon prolonged exposure to mechanical stretching remain, which warrants further investigations.

### Endogenous cues for guiding anisotropic cell organization within hydrogel systems

Unlike exogenous cues, endogenous cues predominantly depend on the cells’ inherent capacity to sense and respond to internally provided cues. This approach emphasizes the cells’ capability to self-align in a specific direction by harnessing the forces exerted during cell-mediated contraction phenomena within hydrogels or through interactions with molecules embedded within 3D hydrogels, creating predefined pathways for guided cellular self-alignment. In comparison to exogenous cues, endogenous ones offer a higher degree of control over the cellular alignment and can be applied to thin and thick hydrogel-based constructs, exhibiting either simple or complex geometrical features. Hydrogel tethering and molecule patterning and leaching are the most widely used endogenous cues for directing muscle cell alignment within hydrogel systems.

#### Hydrogel tethering

Recently, the concept of hydrogel tethering has been employed to engineer 3D tissues with an anisotropic cellular organization [166–168]. This technique is designed to recreate the *in vivo* musculoskeletal microstructure by mimicking the passive force transport between muscle and bone through a connecting tendon [5, 169, 170]. Generally, the cell-laden hydrogel is cast within a mold that contains parallelly positioned pillars [4]. When the casted hydrogel starts to undergo rapid cell-mediated contraction, the pillars restrict this contraction, which builds up a uniaxial tension along the hydrogel’s matrix between the pillars that facilitates the rearrangement of its fibrils into aligned fibers pointing toward the direction of tension [4]. Thus, guiding the alignment of the encapsulated cells toward the same direction of tension (Figure 3D).

To date, the concept of hydrogel tethering has been employed to guide the alignment of various cell phenotypes within hydrogel systems, including muscle cells, ultimately resulting in engineered 3D tissues with organized cellular structures [166–168]. Compared to other techniques, this approach has the advantage of being less aggressive toward the cells and maintaining the built-up cellular alignment throughout the entire culture time because the construct is being tethered. Thereby ensuring a continuous restriction of the hydrogel’s cell-mediated contraction [167]. In addition, the self-guided cellular alignment within tethered hydrogel systems is robust, with complete cellular alignment achievable as early as Day 2 of culture. Agrawal *et al.* [171] assessed the effectiveness of such an approach, casting a cell-laden hydrogel composed of C2C12 and GelMA within a mold having two parallel pillars as tethering points. Robust cellular alignment was achieved within the tethered hydrogel as early as Day 2 of culture, ultimately leading to the development of a thick construct on Day 5 due to the continuous hydrogel compaction. Moreover, the self-aligned cells could fuse into highly aligned and densely packed myotubes by Day 12, as confirmed by immunofluorescence analysis. Similarly, Madden *et al.* [172] encapsulated human primary myoblasts in a hydrogel solution composed of fibrinogen-Matrigel. The cellular hydrogel was dispensed in a silicon mold and anchored between nylon frames as tethering points. Two weeks post-differentiation, the tethered construct contained highly aligned and striated myofibers.

Besides simply casting a cell-laden hydrogel between two parallel pillars in a mold, the pillars may also be incorporated within 3D SMCs of complex geometries during or post-processing [4]. For example, Kim *et al.* [173] produced multilayered fibrinogen-based SMCs via 3D bioprinting to treat excessive VML injuries. To induce the alignment of the incorporated human muscle progenitor cells (hMPCs) within the bioprinted construct, supporting PCL pillars were simultaneously co-printed at the distal and proximal ends of the construct during the bioprinting process. The presence of the pillars at either end of the construct induced the cells to rapidly self-align along the construct’s longitudinal axis upon hydrogel compaction. In a different approach, Choi *et al.* [174] bioprinted hydrogel-based human SMCs, after which they were anchored from either end using metallic pins, which induced rapid cell alignment upon hydrogel compaction over time.

In addition to acting as constraints for the hydrogel’s cell-mediated contraction, the pillars can also be employed to measure and quantify the active forces generated by the engineered muscles as a function of pillar displacement over time [4]. Conventionally, active forces generated from engineered muscles are often measured by harvesting the constructs from the culture dish, followed by placing them in a chamber containing force transducers [175, 176]. Although precise, this method is complicated, invasive, possesses much variability and inconsistency in the results, can damage the muscle construct during the harvesting and mounting process and necessitates the utility of new samples at every timepoint, as tested samples cannot be brought back to culture and used for later timepoints. To overcome these limitations, the pillar system was employed, enabling *in situ* (while anchored between the two pillars) and noninvasive quantification of the contractile forces generated by the engineered SMCs over the course of the study [177]. This is often accomplished by fabricating the pillars using a fixable material [e.g., polydimethylsiloxane (PDMS)], which enables them to undergo deflection during SMC contraction. The contraction-mediated pillar deflection is then used to quantify the maximum contractile force generated by the SMC via ‘pillar-deflection analysis’ [177]. This analysis is done by recording a movie of the deflection and utilizing the movie to measure the maximum pillar displacement post-deflection in pixels using computational scripts [177]. Next, the pillar displacement pixel values are converted into absolute movements in microns using a pixel-to-distance conversion factor in the ImageJ software [177]. Finally, the pillar displacement numbers are converted into absolute contractile forces generated by the SMCs using a force-displacement relationship [177]. Following this method, the maximum contractile forces generated by each SMC can noninvasively be measured and quantified *in situ* during spontaneous and electrically and biochemically induced contractions [177]. Vandenburgh *et al.* [178] were the first to assess the efficacy of such a method to quantify active forces generated from engineered muscles. Here, primary mouse myoblasts were encapsulated in a fibrinogen-based hydrogel. The cellular hydrogel was then cast in a PDMS mold consisting of two parallel pillars. Following 7–8 days, the tethered construct contained highly aligned and sarcomeric myofibers. Moreover, employing a mechanical model made it applicable to convert the pillar displacement into forces produced by the tethered muscles upon electrical stimulation. In a different study, Afshar *et al.* [177] used a similar approach to noninvasively quantify the forces generated by human muscle constructs engineered between two parallel and fixable pillars.

#### Molecule patterning and leaching

Molecule patterning and leaching is an alternative technique to induce cell alignment within hydrogel systems. This approach is based on patterning molecules in 3D hydrogels, which create predefined pathways for cell self-alignment upon the leaching of the patterned molecule (Figure 3E). Currently, this technique has only been shown to be compatible with 3D bioprinting technology. In brief, the molecules are first incorporated within a cell-laden bioink and the resultant mixture is printed layer-by-layer. Due to the intrinsic strain applied during the extrusion process, the bioink undergoes physical stretching, which helps the incorporated molecules to pattern toward the same direction of printing. After printing, the bioprinted construct is washed with a pre-warmed buffer to leach out the patterned molecules. This creates spatially distributed and aligned void structures within the printed construct that act as direct physical cues for guided cell alignment. To date, only one study has utilized this technique to induce muscle cell alignment within hydrogel systems [179]. Here, PVA was incorporated within a cell-laden bioink in a 1:1 ratio, and the resultant mixture was processed for 3D bioprinting. PVA with different molecular weights (MW) (low, medium and high) was incorporated within the cell-laden bioink to investigate the effect of the PVA MW on the overall rheological behavior, physical properties and structural stability of the bioprinted construct as well as viability and alignment of the incorporated cells. It was found that the rheological properties of the bioink were significantly decreased with the low- and medium-MW PVA, while they increased upon the addition of the high-MW PVA. After PVA leaching, the bioprinted construct displayed aligned void structures with the medium- and high-MW PVA, while the low-MW PVA induced randomly aligned void structures. However, the physical properties of the bioprinted construct were not significantly changed with the PVA with different MWs. Further physical examination indicated that the bioprinted construct with the medium- and high-MW PVA presented more stable lattice structures than low-MW PVA. Cell viability analysis revealed that the low- and medium-MW PVA constructs showed significantly higher cell viability than the high-MW PVA. However, F-actin staining showed that the incorporated cells displayed better overall self-alignment in the constructs with the medium-MW PVA compared to the rest of the groups. As a result of this guided self-alignment, the cells could fuse into aligned multinucleated myotubes over time. These data indicate that molecule patterning and leaching approach can be used as a novel and viable method to induce muscle cell self-alignment within hydrogel systems.

The uniqueness of this technique over others lies in its ability to guide the alignment of the incorporated cells toward multiple directions within the same construct. This can be beneficial when attempting to recreate the microstructural organization of muscles that naturally exhibit heterogeneous cellular orientations (e.g., trapezius muscle) [180]. However, this approach is not without limitations. For instance, in comparison to hydrogel tethering, this technique is only compatible with 3D bioprinting technology, which limits its application in various hydrogel-based fabrication methods. In addition, robust cell alignment is typically only observed after 14–21 days in culture. Yet, the overall cellular alignment at these later time points is often not as high as that observed within tethered constructs. Moreover, a considerable number of cells may be lost during the PVA leaching process [181]. Therefore, additional studies are necessary to optimize this innovative technique for expediting and enhancing overall cellular alignment and engraftment. This could involve exploring different materials, utilizing alternative molecules and adjusting material-to-molecule ratios.

## Hydrogel-based strategies for engineering SMCs

Strategies for engineering hydrogel-based skeletal muscle cells (SMCs) aim not only to recapitulate the structural organization of cells within the construct’s design but also to create full-scale and transplantable tissue constructs. These constructs should be capable of filling various defect geometries and enabling immediate structural and functional repair upon transplantation. Such strategies are designed to precisely control essential features of the engineered construct, such as overall geometries, fiber diameter and pore size and architecture. To date, several biofabrication strategies have been developed and utilized for the engineering of hydrogel-based SMCs, including cast molding, electrospinning, 3D bioprinting, ejection and microfluidic spinning (Figure 4). This section will discuss these biofabrication strategies and highlight their advantages and limitations in the context of VML treatment, as summarized in Table 4.

**Figure 4:**
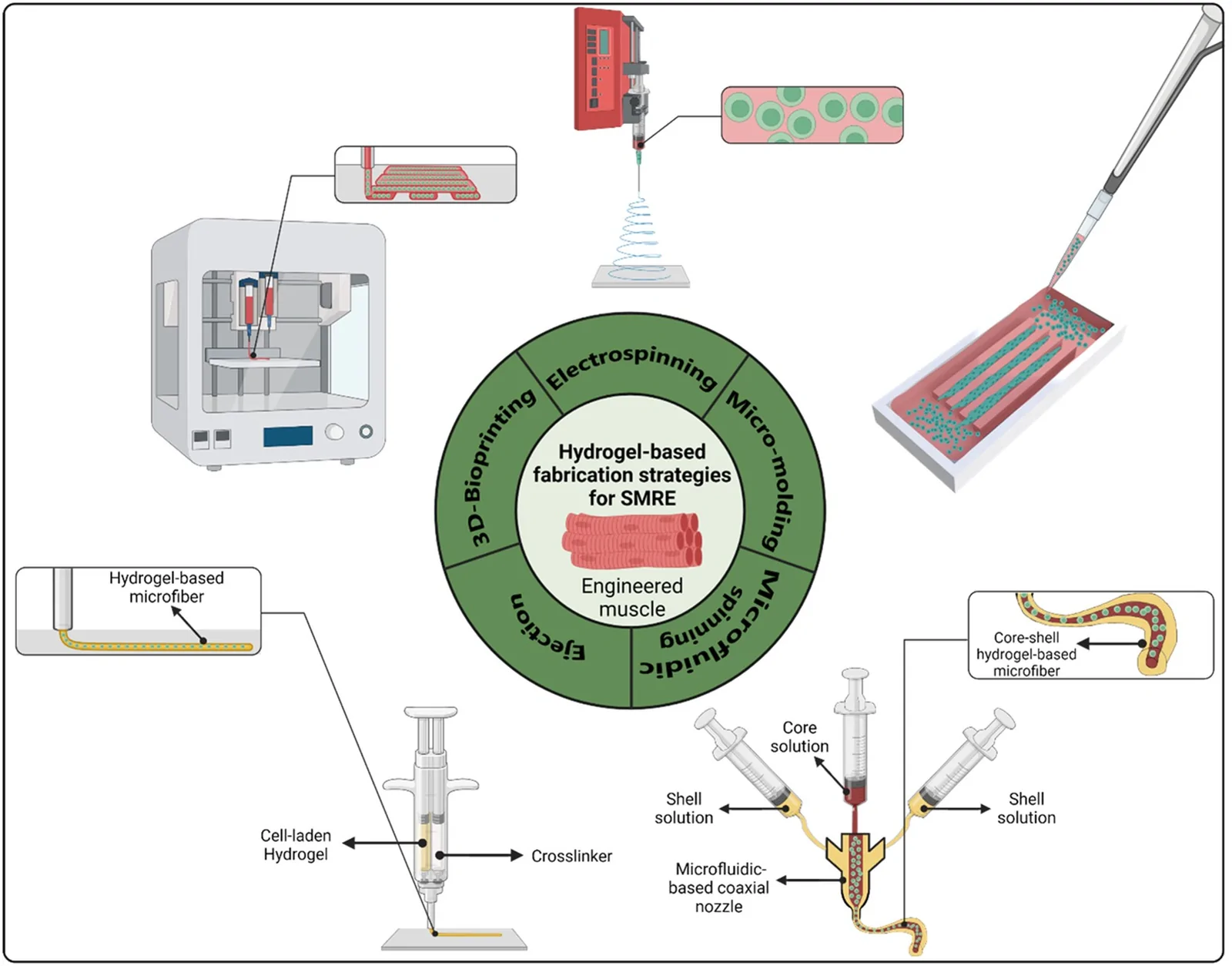
Schematic representation of the various hydrogel-based fabrication strategies employed in SMRE.

**Table 4:** Current hydrogel-based strategies employed for engineering SMCs, along with the advantages and disadvantages of each strategy.

Strategy	Pros	Cons	Reference(s)
Cast molding	Simple Fast Cost-effective Compatible with all hydrogels Can produce constructs with muscle-like bundle structures.	Unable to produce constructs with complex architectures. Unable to produce large-scale or multi-layered constructs for human indications.	[182–191]
Cell electrospinning	Simple Cost-effective Can produce constructs with fibrous architectures resembling the fibrous structure in the skeletal muscle.	Can be time-consuming, depending on the size of the construct Can significantly affect cell viability Not compatible with all hydrogels unless chemically modified or used in high viscosities. Pore size cannot be controlled	[192–207]
3D bioprinting	Can produce constructs with defect-specific geometries and complex architectures. Can achieve constructs with spatial arrangements of different materials and cell phenotypes. Can precisely control the pore size.	Expensive Complicated Can affect cell viability due to the shear forces experienced during bioink extrusion Requires more cells than in other approaches Cells suspended in the bioink can potentially undergo gravity-mediated sedimentation during prolonged printing, affecting their homogenous distribution in the resultant construct. Very time consuming (requires several hours to days to produce large-scale constructs for human indications) Not compatible with all hydrogels unless chemically modified or used in high viscosities.	[44, 173, 208–219, 222–226]
Ejection	Simple Fast Cost-effective Can generate cm-long hydrogel microfibers	Unable to recapitulate the 3D complexity of natural tissues in the resultant construct The produced fibers need to be grouped in bulk to recreate full-scale constructs, which can induce a hypoxic construct’s core due to the lack of a well-defined pore volume. Not compatible with all hydrogels unless chemically modified or used in high viscosities.	[227–230]
Microfluidic spinning	Simple Fast Cost-effective Can generate cm-long hydrogel microfibers. The diameter of the fiber can be highly controlled	Unable to recapitulate the 3D complexity of natural tissues in the resultant construct The produced fibers need to be grouped in bulk to recreate full-scale constructs, which can induce a hypoxic construct’s core due to the lack of a well-defined pore volume. Not compatible with all hydrogels unless chemically modified or used in high viscosities.	[36, 231–238]

### Cast molding of hydrogels

Cast molding is an easy, fast and cost-effective strategy to produce SMCs [182–184]. It is based on casting a cell-laden hydrogel into a mold with pre-defined geometries and architectures, followed by crosslinking the cast hydrogel to attain the mold’s negative shape [184]. PDMS is a widely utilized material for molds fabrication because of its biocompatibility, transparency, elasticity, mechanical strength, ease of processing, tunable curing rate and the ability to infiltrate into microscale and nanoscale mold features [151, 185, 186]. An additional advantage of utilizing PDMS for mold fabrication is the ability to easily detach the crosslinked hydrogels from PDMS-based molds with minimal to no structural deterioration thanks to its hydrophobic nature [186]. In addition to PDMS, other materials such as polytetrafluoroethylene (PTFE), poly(methyl methacrylate) (PMMA), glass and metals have also been utilized to fabricate molds [151,152, 185, 187]. Moreover, due to the recent advancement in additive manufacturing technology, molds with complex features could now be obtained using a large library of stiff and elastic resins, enabling the engineering of SMCs with much more complex architectures [152]. Furthermore, sacrificial materials such as PVA and gelatin have also been considered and utilized to fabricate molds, offering new means for mold fabrication [188, 189]. Molded hydrogels can be crosslinked by several methods, including thermal, physical, enzymatic and ultraviolet (UV)-photocrosslinking. Therefore, the cast molding technique can use hydrogels with different cross-linking modes.

To date, molds with different design geometries, such as channel or cylindrical-like cavities, have been produced, allowing the construction of SMCs with multi-bundle structures [7, 163]. Also, the cylindrical cavities within such molds can be confined to produce SMCs with different bundle diameters, enabling studying the bundle diameter’s effect on muscle cell alignment. For instance, Costantini *et al.* [190] injected a cell-laden hydrogel composed of C2C12 and GelMA into a PDMS-based mold consisting of multiple channels of the same length. In this study, a SMC with a multi-bundle structure was obtained post-UV-crosslinking, which was then anchored from both ends to induce cell alignment upon hydrogel compaction. After 2 weeks of culture, it was found that the C2C12 cells could align along the bundles’ longitudinal axis and fuse into aligned multinucleated myotubes based on the immunofluorescence analysis. Next, the same mold was re-produced but with different channel diameters (2000-µm, 1000-µm and 500-µm) to study the influence of the geometrical confinement on myotube orientation. The results showed that by reducing the channel diameter, the packing and alignment of the formed myotubes were enhanced and better resembled the morphological structure of the native tissue. Besides myotube orientation, the channel diameter has also been shown to influence the SMC’s overall contractile force production. Morimoto *et al.* [191] produced three PDMS molds with different channel diameters (1000-µm, 750-µm and 500-µm). Rat muscle progenitor cells (rMPCs)/Matrigel-fibrinogen solution was dispensed into the mold, and the SMCs were formed once enzymatic crosslinking with thrombin was achieved. To induce cell alignment, the resultant SMCs were pinned at both ends. After 4-days of culture, the SMCs with a bundle diameter of 500-µm contained more closely packed and aligned myotubes compared to the SMCs produced with bundle diameters of 750-µm and 1000-µm. As a result of this high myotube packing and alignment, the SMCs with a bundle diameter of 500-µm generated significantly higher contractile forces upon electrical stimulation compared to the SMCs with the larger bundle diameters. Further analysis revealed that the contractile force generated by the SMCs with a bundle diameter of 500-µm was approximately 2- and 2.5-fold higher than the ones generated by the 750-µm and 1000-µm SMC groups, respectively.

Although the cast molding approach could produce SMCs consisting of muscle-like bundle structures with high cellular organization, it only enabled micron-scale tissue or single-layered muscle bundle construction that may not be applicable for treating extensive muscular defects. Thereby lacking the potential for future translation into humans [4]. In addition, while assembling the resultant single-layered constructs may seem a viable approach to reconstructing full-scale 3D SMCs, it may not be feasible in clinical settings due to potential structural deterioration or contamination during the assembly process.

### Electrospinning of hydrogels

Electrospinning is a relatively old technique employed to produce microscale and nanoscale fibrous structures from a starting polymeric solution. This technique dated back to the early 1600 s and was mainly used to produce fibrous membranes employed as air/water filters [192–195]. However, the discovery that the ECM’s microenvironment is constituted of fibrous structures similar in scale, size and morphology to the ones observed in electrospun products has dramatically changed the course of this technique [196]. Since this discovery, electrospinning has been widely employed in biomedical research to generate biomimetic structures as wound dressings and, more recently, as substrates for tissue regeneration [197]. The typical electrospinning setup consists of a syringe with a metallic needle, a syringe pump, a high direct current (DC) voltage generator and a grounded collector [197]. The polymeric solution is first loaded into the syringe, followed by placing the loaded syringe in the syringe pump. During the process, a high voltage is applied to the metallic needle, which overcomes the surface tension of the ejected polymeric solution [197]. This causes the ejected polymeric solution to flow in the direction of the electrical field. A further increase in the electrical field causes the ejected solution to assume a conical shape (Taylor cone) [197]. At this point, ultrafine nanofibers emerge from the Taylor cone, which are then collected on the grounded collector. By optimizing specific parameters such as flow rate, the applied electrical current and the distance between the needle tip to the grounded collector, fibers in the size range of sub-microns down to the nanoscale can be produced [197]. In addition, depending on the method of collection, nanofibers in random and aligned arrangements can be obtained [198]. For SMRE applications, nanofibers with the aligned arrangement are favored to ensure high levels of cellular organization [199, 200]. To obtain electrospun nanofibers with aligned architecture, the polymeric solution is directly deposited on a high-speed rotating mandrel [200]. Optimizing the speed of rotation can achieve a high degree of alignment. Typically, higher rotating speeds result in a greater alignment of fibers being obtained.

Synthetic materials such as PLA, poly(lactic-co-glycolic acid) (PLGA) and polycaprolactone (PCL) have frequently been used to produce electrospun fibrous structures for SMRE due to their high spinnability and the unnecessity of crosslinking them post-fabrication in comparison to natural materials [199]. However, synthetic materials possess several limitations, including low biocompatibility, toxic degradation by-products and the lack of cell-adhesion sites for cell attachment [201]. To improve the biological performance of synthetic electrospun nanofibers, attempts have been made to composite synthetic materials with natural ones (e.g., collagen and chitosan) [202]. However, regardless of the type of the starting material, the conventional electrospinning method requires manually seeding the cells on the resultant structure to produce the SMC, which presents several disadvantages. The first limitation is the difficulty in achieving a uniform cellular distribution across the surface of the scaffold, which can result in suboptimal myoblast proliferation and, thereby, poor fusion rates. Further, the penetration of the manually seeded cells to deeper scaffold regions often depends on the scaffold’s porosity. Scaffolds with an aligned nanofiber arrangement are known to exhibit significantly smaller pore sizes compared to scaffolds with a random nanofiber orientation [203]. This can prevent the seeded cells from infiltrating into the deepest regions of the scaffold which can affect the overall outcomes upon transplantation *in vivo* [203]. Moreover, manual seeding necessitates incubating the seeded scaffold for some time (often for 1–2 h) before media addition to allow the seeded cells to attach partially. This may lead to the dehydration of a significant portion of the seeded cells, thereby impacting the overall viability of the cells. To overcome these limitations, recent advancements in biofabrication technology enabled cell-electrospinning (CE). This modified approach allows premixing the cells into the hydrogel prior to electrospinning, thus ensuring a homogeneous cellular distribution within the resultant construct [204]. While relatively new, CE was explored in SMRE. Yeo and Kim [205] were the first to employ this technique to produce bead-free electrospun SMCs with a homogeneous cellular distribution. Here, C2C12 were ladened within alginate/polyethylene oxide (PEO) and electrospun to produce an electrospun construct with aligned fiber arrangement. To ensure high cell viability after the electrospinning process, a relatively low voltage was used which resulted in retaining the cell viability. Moreover, the aligned electrospun alginate fibers guided high cellular alignment which subsequently differentiated into aligned multinucleated myotubes by one week of culture. In a separate study, murine myoblasts were loaded into fibrin/PEO solution after which the mixture was electrospun into an aligned fibrous construct [206]. To achieve robust crosslinking, the cell-ladened hydrogel was extruded into a thrombin/calcium chloride (CaCl_2_) rotating bath. By modifying several electrospinning parameters such as applied voltage, flow rate, the distance between the needle tip and the grounded collector, and the electrospinning time, dramatically high cell viability was achieved in the resultant construct. The cell-ladened fibrous structure was cultured for up to 7 days, after which it was assessed for cell growth, alignment and myogenic differentiation. Immunofluorescence images revealed that the encapsulated cells could rapidly proliferate, elongate and differentiate into multinucleated myofibers. Further analysis revealed that the number of myotube-associated nuclei, length and myotube diameter significantly improved over the course of culture.

Although CE seems to be a scalable approach for producing 3D SMCs with a homogeneous cellular distribution and an anisotropic cellular organization, concerns regarding cell viability remain. For instance, to ensure high cell viability during CE, relatively low voltage values are preferable [206]. However, given that some materials require high voltage or, otherwise, they cannot be electrospun (e.g., collagen) [207], the choice of materials can be limited. In addition, due to the need for instant crosslinking during the electrospinning process to preserve shape fidelity, only a few numbers of hydrogels can be used in CE. This is because not all hydrogels can rapidly be crosslinked in nature (e.g., ECM-derived hydrogels, collagen, gelatin and chitosan) unless they are composited with other fast-gelling materials or chemically modified (e.g., through functionalization with methacrylate or a phenol group to produce photo- or enzymatically cross-linkable hydrogels, respectively).

### Three-dimensional bioprinting of hydrogels

Three-dimensional bioprinting is arising as a new tool to produce bioengineered constructs with complex geometries by building up cell-laden hydrogels in a layer-by-layer fashion [208]. With this technology, it is possible to build constructs with patient-specific defect architectures, thus ensuring a solid construct-tissue integration post-transplantation *in vivo* [209]. Generally, the cell-laden hydrogel, or the so-called ‘bioink’, is loaded into the bioprinter’s cartridge in preparation for extrusion [210]. The bioink is then extruded through a nozzle as a continuous cell-laden hydrogel filament. The extrusion process is guided by an applied pneumatic pressure, which its value is often determined based on the bioink’s viscosity [210]. Finally, a full-scale tissue construct can be obtained by depositing the cell-laden hydrogel filament layer-by-layer. Depending on the bioink’s properties (e.g., viscosity and nature of crosslinking), it is possible to predetermined whether the presence or absence of a supporting material is required to achieve constructs with high shape fidelity [210]. In addition, hydrogel filaments with varying diameters can be achieved within the resultant construct depending on the viscosity of the bioink and the diameter of the nozzle used. Due to its ability to recapitulate the tissue’s microenvironment at the macroscale and microscale, 3D bioprinting has been widely used as a potential technique to recreate the 3D configuration of a wide variety of biological tissues, including skeletal muscles [210, 211].

In 3D bioprinting of skeletal muscles, the ultimate goal is to produce SMCs that are structurally stable and possess anisotropic cellular organizations and ultrastructures similar to the native muscle. To achieve this goal, several 3D bioprinting strategies (e.g., indirect, microfluidic-assisted and direct 3D bioprinting) have been attempted chiefly by exploiting the bioinks’ properties, such as gelation mechanism and viscosity. In the indirect 3D bioprinting approach, for instance, a sacrificial supporting material is often co-printed alongside the bioink to maintain its structural integrity until a terminal crosslinking is achieved. Post-terminal crosslinking, the sacrificial supporting material is removed, resulting in a construct with a well-preserved architecture. Kim *et al.* [173] used an integrated tissue-organ printing (ITOP) system capable of printing multiple materials simultaneously. The system was used to fabricate human SMCs made entirely from fibrinogen. However, since fibrinogen exhibits a water-like viscosity, a sacrificial acellular support consisting of gelatin was co-printed alongside fibrinogen to encase it until the crosslinking was achieved. Moreover, supporting PCL pillars were simultaneously co-printed at the distal and proximal ends of the construct during the bioprinting process to maintain shape fidelity and induce cell alignment upon hydrogel compaction. Post-bioprinting, the construct was submerged in a prewarmed media supplemented with thrombin to induce fibrinogen crosslinking and dissolve the gelatin sacrificial support. Dissolving the gelatin away also created microchannels between the cell-laden patterns, serving as pores for oxygen and nutrient diffusion. The bioprinted SMCs were further cultured for 9 days, during which they were subjected to *in vitro* evaluations. To confirm the efficacy of their bioprinting system, mold-cast constructs made using the same material and cell type were included as a control group for comparison. It was found that the bioprinted construct increased the cell alignment compared to the cast-mold constructs. Additionally, they observed that the bioprinted constructs could maintain high cell viability and promote their differentiation into multinucleated myofibers. In contrast, the cells in the cast-molded constructs were mostly dead by Day 5 of culture. Also, upon transplantation *in vivo* in a rat model of VML, they observed that defects treated with the bioprinted constructs contained significantly increased volumes of newly regenerated muscle compared with defects treated with the mold-cast hydrogels with and without cells. In addition, treatment with the bioprinted constructs restored the contractile force by ∼85% at 8 weeks compared to the other groups. Furthermore, the authors observed that the bioprinted construct had integrated with the host’s vascular and neural systems, as evidenced by the immunohistochemistry (IHC) analysis against vascular and neural markers, respectively. Although this study demonstrated an innovative technique to bioprint SMCs with good overall structural integrity and therapeutic potential, the necessity for subjecting the gelatin sacrificial support to dissolution prior to the complete gelation of the fibrinogen cell-laden bioink can potentially lead to the bioink spreading, ultimately resulting in poor filament resolution within the bioprinted construct.

To address this issue, Costantini *et al.* [212] employed a microfluidic-assisted 3D bioprinting approach. This approach often utilizes a bioprinter equipped with a coaxial nozzle. The main advantage of the co-axial nozzle is that both the crosslinking solution and the cell-laden hydrogel can be simultaneously delivered through outer and inner concentric needles [213–216]. This enables the extruded cell-laden hydrogel to undergo instant gelation upon contact with the crosslinker. Thus, preventing the bioink from spreading or collapse during bioprinting, ultimately resulting in an improved filament resolution [213–216]. Therefore, it can be speculated that hydrogels employed in microfluidic-assisted 3D bioprinting should be able to undergo rapid gelation at the tip of the coaxial nozzle upon contact with the crosslinker [4]. To meet such a criteria, the authors developed a bioink composed of PEGylated fibrinogen as the core material mixed with alginate as the sacrificial supporting material. The bioink was ladened with C2C12 myoblasts and subsequently fed into the inner needle of the coaxial nozzle, whereas the outer needle was filled with CaCl_2_. During extrusion, CaCl_2_ encased the bioink, causing instant gelation of the sacrificial alginate component, which maintained the filament’s resolution during the entire bioprinting process. Post-bioprinting, the constructs were exposed to a UV light source to crosslink the PEGylated fibrinogen component of the bioink. Subsequently, the constructs were incubated in an EDTA bath to wash off the alginate sacrificial material. This process resulted in SMCs with excellent shape fidelity and high fiber resolution. The resultant constructs were *in vitro* evaluated for their myogenic potential. Within three to four culture days, the encapsulated myoblasts showed a high degree of alignment. By Day 21 of culture, highly aligned and sarcomeric myofibers were observed. Moreover, upon transplantation in the back of nude mice for 4 weeks, organized muscle formation was evidenced within the bioprinted construct.

In addition to the bioprinting approaches mentioned earlier, the direct 3D bioprinting method can be employed to produce structurally stable and self-standing SMCs. This is achieved without the necessity for sacrificial supporting materials or *in situ* crosslinking by using a bioink with high viscosity. The advantage of using highly viscose bioinks is the ability to maintain the printing resolution before being subjected to a terminal crosslinking, thus, eliminating the need for sacrificial supporting materials or an on-process crosslinking [217, 218]. GelMA is one of the few materials that inherently exhibits a high viscosity, which makes it an optimal candidate for fulfilling this approach [219]. However, maintaining the stability of GelMA-based structures during culture is often challenging due to its poor degradation profile. To address this issue, García-Lizarribar *et al.* [220] blended pristine GelMA with nondegradable synthetic hydrogels in an attempt to achieve stable and durable GelMA-based bioinks. To formulate the bioinks, methacrylated alginate, carboxymethyl cellulose or PEGDA were combined with GelMA, ultimately producing GelMA-alginate-methacrylate, GelMA-carboxymethyl cellulose-methacrylate and GelMA-PEGDA. The different bioinks, including the pristine GelMA, were then ladened with C2C12 and successfully bioprinted into structurally stable and self-standing SMCs. However, on Day 11 of culture, the SMCs made using the pristine GelMA underwent rapid degradation and became flat, while those made using the composite hydrogels maintained their 3D structure. Therefore, the structural stability achieved within the composite hydrogel-based SMCs allowed the cells to proliferate, align toward a single uniaxial direction and subsequently fuse into aligned myotubes.

While 3D bioprinting is by far the most promising biofabrication technique for creating hydrogel-based constructs with complex architectures, it exhibits several limitations that need further research attention. For instance, in addition to its high cost, time consumption and the need for obtaining highly expert personnel to operate the bioprinter, 3D bioprinting significantly affects the viability of cells due to the shear stresses experienced during the extrusion process, and the cell viability can drop to as low as 40% after bioprinting [221]. Furthermore, due to the poor maintenance of cell viability during bioprinting, high initial cell densities (30–50 million cells/mL) are often required for 3D bioprinting [173]. In this sense, the need for high cell densities can be problematic in clinical settings because the volume of the tissue biopsy obtained from patients is often limited and may yield an insufficient number of cells for 3D bioprinting, especially if large muscle constructs need to be printed [44, 222].

Another limitation of 3D bioprinting is that it often requires the utility of highly viscous bioinks to ensure shape fidelity maintenance during printing [217, 218]. Yet, highly viscous hydrogels are unfavorable to cells because they can restrict their movement, spreading and migration, thus resulting in cell death and poor tissue formation. Additionally, due to the perpendicular orientation of the bioink cartridges, most of the suspended cells within the bioink can potentially undergo a gravity-mediated sedimentation [223–225]. This can result in uneven cell distribution within the bioink, ultimately leading to undesired cell agglomeration, poor viability or insufficient tissue formation at different regions of the printed construct [223–225]. Moreover, not every hydrogel is printable, and only those that meet the specific printing requirements, such as hydrogel viscosity, nature of crosslinking and gelation speed, can be 3D printed [226].

### Ejection of hydrogels

Hydrogel ejection is a recent yet simple and cost-effective technique for generating cm-long hydrogel microfibers. To create the microfiber, the hydrogel is first loaded into an ejection apparatus such as a syringe or a micro-nozzle array [227, 228]. The microfibers are then obtained by directly ejecting the hydrogel into a bath containing the crosslinking agent or directly extruding a pre-crosslinked hydrogel. For reconstructing full-scale 3D constructs, the resultant microfibers are often grouped into a bulk structure after orienting them in an aligned manner. Therefore, the use of microfibers as building blocks is a straightforward technique to reconstruct tissues with hierarchical structures. Due to its simplicity and ability to recapitulate the micro-organization of fiber-shaped tissues, hydrogel ejection has been utilized as an approach to produce hydrogel-based SMCs.

Li *et al.* [229] were the first to attempt to use hydrogel ejection as a strategy to produce SMCs. Here, the authors fabricated cell-laden hydrogel microfiber-based constructs, named hydrogel noodles, inspired by the fabrication approach used to produce the Chinese Hele noodles. To fabricate the construct, C2C12 were first ladened within a solution of GelMA-poly(ethylene glycol) dimethacrylate (PEGDMA) (GelMA-PEGDMA), loaded into a syringe and then photo-crosslinked by exposure to a UV light source. After cross-linking, the cell-laden hydrogel was squeezed through a sieve to fabricate Chinese noodle-like microfibers. This process enabled the generation of hundreds to thousands of hydrogel microfibers within seconds. In addition, the authors could control the diameter of the microfibers by ejecting the hydrogel through sieves of different pore sizes. This approach allowed obtaining microfibers in a diameter range of 129-µm and 300-µm. The resultant microfibers were then subjected to initial *in vitro* assessments which focused on evaluating the effect of the ejection technique on C2C12 viability. The results revealed that the cells were highly viable (<90%) within the microfibers ejected using both pore-size sieves, suggesting that the ejection process did not negatively affect the cells’ viability. Next, the constructs were subjected to a uniaxial mechanical stretching, which could guide the alignment of the encapsulated cells toward the direction of stretching and improve their proliferation and fusion into aligned myotubes compared to the constructs receiving no mechanical stretching.

Hydrogel ejection can also be employed to produce complex cell-laden fiber structures, which can be achieved via integrating the ejection apparatus to a kinetics static mixer (KSM) [230]. Bolívar-Monsalve *et al.* [230] used this method to produce cell-laden hydrogel fibers that spatially mimic the compositional complexity seen in the skeletal muscle fascicle. In brief, C2C12 were first suspended in a hydrogel solution composed of GelMA-alginate. The cell-laden hydrogel solution and an acellular pristine alginate hydrogel solution were then loaded into KSM’s different compartments and co-ejected simultaneously into a CaCl_2_ bath to crosslink the alginate. Next, the resultant fibers were subsequently exposed to a UV light source to photo-cure GelMA. This process resulted in the generation of a homogenous filament that exhibited eight inner intercalated micro-layers of the cell-laden GelMA and pristine GelMA, thus resembling the critical structural features of the native muscle fascicle. *In vitro* evaluations of the generated fibers revealed that the encapsulated cells were highly viable (∼90%) at Day 7 and continued to be viable until Day 28 (∼80%). Interestingly, cross-sectional analysis showed that the cells did not migrate to neighboring layers during culture, which confirmed that the intercalated layers containing only pristine alginate could effectively serve as a physical barrier at the microscale and confine cells to the striation in which they were initially seeded. Further *in vitro* analysis showed that the confined cells were able to fuse into highly aligned and densely packed myosin heavy chain (MHC) and sarcomeric α-actin (Sa-α) positive myofibers.

Despite being much simpler than many other techniques and its ability to yield a large volume of microfibers within a short period, the hydrogel ejection approach displays several limitations that can hinder its ability to generate volumetric SMCs suitable for treating VML. For instance, unlike 3D bioprinting, the 3D complexity of the natural tissues cannot be recapitulated using this approach. Moreover, the resulting cell-laden hydrogel microfibers need to be densely packed into a fibrous structure to recreate full-scale constructs. However, this strategy may lead to the generation of hypoxia at the core of the construct due to the insufficient or lack of pre-established pore volume. Hence, the viability of constructs produced through this approach for treating extensive VML defects, particularly in humans, remains uncertain. Another limitation is the restricted selection of hydrogel materials compatible with this method, given the requirement for hydrogels with instantaneous crosslinking properties.

### Microfluidic spinning of hydrogels

Microfluidic spinning has emerged as a potential tool for generating cm-long microscale and nanoscale hydrogel fibers in a relatively short time without the need for using complicated devices or facilities [231]. Similar to the hydrogel ejection approach, full-scale 3D constructs can be obtained by grouping the resultant hydrogel fibers into a bulk fibrous structure [36, 231]. However, a key difference between both approaches is that in microfluidic spinning, the hydrogel solution flows out of the syringe through microfluidic channels before being extruded. This key difference has many advantages, including the ability to produce hydrogel fibers with a little starting hydrogel volume and precisely controlling and manipulating the diameter of the extruded fiber [34]. Due to these unique qualities, microfluidic spinning has been used to fabricate various tissues that inherently exhibit fibrotic microstructures, including skeletal muscles [232–234].

Indeed, hydrogels with fast crosslinking properties are required for microfluidic spinning to ensure the maintenance of shape fidelity upon extrusion. In this context, alginate is widely used for microfluidic spinning thanks to its instant ionic gelation properties [235]. A common strategy to fabricate alginate-based fibers is by simultaneously exposing the alginate to CaCl_2_ upon extrusion, which is often achieved by employing a coaxial needle. Costantini *et al.* used a coaxial needle embedded within a microfluidic device to generate cell-laden PEGylated fibrinogen/alginate-based microfibers [236]. Murine and human cells were first suspended within the hydrogel solution and the cell-laden hydrogel was simultaneously co-extruded with CaCl_2_ to achieve an instant alginate crosslinking. Upon extrusion, a hydrogel blob was formed at the tip of the coaxial nozzle, which was gently pulled with a tweezer, forming a hydrogel fiber, until it reached the surface of a rotating Teflon drum for collection [235]. The collection process continued until a bundle of highly packed microfibers was obtained, mimicking the anisotropic muscle organization within the resultant construct. Finally, the resultant constructs were UV crosslinked to stabilize the PEG-fibrinogen component of the hydrogel solution. Optimizing the hydrogel solution’s flow rate and rotating speed made it possible to achieve a fiber resolution of 80–100 µm, which often cannot be attained following the simpler hydrogel ejection approach. The fabricated constructs were further evaluated *in vitro* for cell alignment and myofiber formation. The same cell-laden hydrogel solution was used to produce a bulk hydrogel structure as a control group. After 2 weeks of culture, highly aligned and densely packed myofibers were observed in the fibrous constructs, whereas the bulk hydrogel contained randomly aligned myofibers. Moreover, the efficacy of the fibrous construct to treat excessive muscle injuries was examined in a murine tibialis anterior (TA) VML defect *in vivo*. They found that defects treated with the fibrous constructs contained significantly increased volumes of newly regenerated muscle at week 2 post-transplantation compared with defects receiving no treatment. In addition, the contractile force improved substantially following treatment compared to the defect control. Furthermore, the host vascular and neural integration to the transplanted construct were observed, as confirmed by IHC analysis against vascular and neural markers, respectively.

No doubt, alginate is a suitable material for microfluidic spinning due to its intrinsic gelation and mechanical properties, which guarantee preserving the shape of the extruded fiber. However, alginate lacks cell adhesion peptides and cannot be degraded in mammalian systems because it is sourced from plants [235]. Therefore, its clinical translational potential is poor. While natural materials offer a better option, their sole utility for microfluidic spinning is not possible due to their prolonged gelation time and low mechanical properties. To solve this issue, Onoe *et al.* [237] developed an innovative microfluidic-based approach to enable the spinning of natural materials such as collagen with high shape fidelity. By utilizing a coaxial needle comprising of C2C12/collagen solution in the core and alginate/CaCl_2_ in the sheath, it was possible to produce collagen microfibers sheathed by alginate. Such an approach encased the cells and the natural material in the core of the fiber, thus, preventing it from spreading during the extrusion process. After subjecting collagen to thermal gelation at 37°C, the alginate sheath was dissolved away by incubation in an alginate-lyase bath, resulting in microfibers made entirely from collagen. The resultant microfibers were evaluated for their myogenic potential, revealing increased myotube formation over time.

In a relatively similar approach, Wang *et al.* [238] used PVA instead of alginate as the sacrificial material to ultimately yield C2C12-GleMA/gelatin hollow microfibers with high shape fidelity. Here, the microfibers consisted of a PVA core sheathed with the cell-laden hydrogel. Both solutions were loaded into a microfluidic co-axial needle, then directly and simultaneously co-extruded into a cold phosphate buffer saline (PBS) bath to ensure rapid physical solidification of the gelatin component. Subsequently, gelation of the GelMA/gelatin solution rapidly occurred by thermo-crosslinking once in contact with the cold PBS, and irreversible photo-crosslinking was further obtained by exposure to a UV light source. Finally, once the GelMA and the gelatin components of the cell-laden hydrogel were completely crosslinked, the resultant microfibers were incubated in a PBS bath at 37C to wash off the PVA from the microfiber’s core. This process resulted in the generation of hollow microfibers made entirely from a natural material with high structural stability. Furthermore, subsequent *in vitro* evaluations revealed that the produced microfibers could sustain high cell viability over time.

Although microfluidic spinning has many advantages over other techniques, such as simplicity and the ability to obtain cm-long hydrogel-based microfibers within a relatively short time, it suffers from the same limitations found in the hydrogel ejection approach, particularly the unraveled capability of the constructs produced using this approach for treating large-scale VML defects. Therefore, the utility of this approach to histologically and functionally treat large-scale VML defects should be vigorously investigated in large animal models to predict its suitability for curing extensive muscle defects in humans.

## Conclusion and scalability considerations for clinical translational purposes

At present, autologous muscle transfer stands as the gold standard among various clinical approaches for treating VML [18–20]. Despite the imperative to explore alternative treatment methods due to its notable drawbacks, this approach has demonstrated substantial muscle regeneration and favorable functional outcomes [1, 21, 22]. Autologous muscle grafts possess essential attributes, including viable, mature, contracting, densely packed and aligned myofibers, an established ECM framework, intact vascular and neural networks and a substantial reserve of quiescent SSCs. These factors collectively contribute to the instant muscle recovery and effective functional restoration observed with this approach. Consequently, the development of engineered skeletal muscle tissues that replicate all facets of native muscles emerges as a promising alternative, potentially overcoming the existing drawbacks associated with autologous muscle transfer.

Although the foundation of SMRE was established on the utility of synthetic materials-based scaffolds, the future of SMRE may dramatically depend on the combination between cell-laden hydrogel systems and biomimetic fabrication techniques to ultimately achieve engineered SMCs that resemble not only the native skeletal muscle structure and cellular organization but also its mechanical resilience and viscoelastic properties. While considerable efforts have been made in the past several years in establishing hydrogel-based fabrication techniques to guide the anisotropic cellular organization within hydrogel systems and process them into full-scale 3D constructs with several architectures and defect-specific geometries, the field is still in its infant stage.

Indeed, the most vital requirements for any hydrogel-based fabrication strategy toward skeletal muscle engineering are the ability to recreate the cellular organization and the 3D volume of the native tissue in the resultant construct [170]. While these essential fabrication requirements have so far been met in almost all of the previously established biofabrication methods, size scalability is a crucial yet neglected criterion. The ultimate goal of SMRE is to develop translational technologies that can encourage muscle regeneration following trauma to address the high unmet demands of patients [28–30, 239–244]. No doubt, evaluating the potential of novel technologies toward muscle regeneration and repair in small lab-scale settings (e.g., small-sized constructs in small animal models) is essential, can provide valuable preliminary information on their efficacy, and should not be disregarded. However, bearing in mind the translational potential of the developed technologies during the early-research planning stage is as important and can fasten their translation from the bench-top to the clinic. Regrettably, according to the current review, most studies, if not all, tend to focus their efforts on addressing fundamental research questions (such as material properties, fabrication-related material compatibility, the level of structural complexity and cellular organization achievable through a newly developed fabrication method) rather than on the scalability and translational aspects of the developed technologies, even though both aspects are equally crucial. For instance, cell electrospinning, hydrogel fiber ejection and microfluidic spinning are all efficient fabrication methods that can produce fibrous constructs with great cellular organization. In addition, many hydrogels have been used in their pristine form or modified to increase their compatibility with these methods. However, the ability of these approaches to produce large-scale constructs for human indications is not feasible due to the lack of a pre-established and well-defined pore volume that can allow for oxygen diffusion to the deepest layers of the constructs for meeting the high metabolic demands of cells. Hence, balancing between the basic research questions and clinical translational potential during the early-stage research can lead to increasing the number of products in the clinic, which is the ultimate goal of RE.

In general terms, for a hydrogel-based fabrication strategy to be considered scalable, it should demonstrate the capability to produce identical constructs in various sizes, scaling up to the human scale (cm^3^), without inducing hypoxia at the core of the construct. In this context, it has previously been reported that muscle cells can tolerate low oxygen levels (<1%) for a maximum duration of 1.5 h [245]. After this timeframe, irreversible hypoxia can be induced, ultimately leading to cell apoptosis. It is important to note that hypoxia can be induced at the core of the construct during the fabrication process, throughout the culture period, or even after transplantation. In this context, we aim to elucidate the conditions leading to hypoxia generation in the core of hydrogel-based constructs in each of these scenarios and propose potential solutions to mitigate these effects.

### Hypoxia generation during the fabrication process and potential solutions

For instance, in comparison to the hydrogel-based biofabrication methods discussed here, 3D bioprinting exhibits a higher scalability potential due to its ability to establish a well-defined pore volume within the resultant construct, which can aid in the continuous diffusion of oxygen. In this frame, the most efficient method to preserve the structural integrity and pre-established pore volume within 3D bioprinted constructs is by directly depositing the bioink into a sacrificial supporting bath (e.g., gelatin bath) [173,174, 246]. Therefore, the utility of a sacrificial supporting bath is often preferred during 3D bioprinting. However, due to the lack of a pore volume within the sacrificial bath, the diffusion of oxygen into its deepest regions may be severely limited during the fabrication process. This suggests that to prevent a hypoxic construct’s core, the entire fabrication process should not exceed the timeframe reported for hypoxia induction in muscle cells. Although SMCs with hypoxia-free cores were successfully bioprinted within sacrificial supporting baths [174], their sizes have so far never exceeded the lab scale. Therefore, the time required to produce such small constructs never exceeded the hypoxia induction timeframe limit. In cases where larger constructs are needed for human indications, the fabrication time may dramatically increase beyond the timeframe limit (e.g., 868.05 h for a construct with dimensions: *W*: 10 × *H*: 15 × *D*: 5 cm^3^ at a typical printing speed of 160 mm/min) thus increasing the risk of hypoxia generation at the construct’s core. Therefore, more studies are required to estimate the scalability of 3D bioprinting for producing large-scale (cm^3^) SMCs with hypoxia-free cores.

No doubt, 3D bioprinting is shaping the future of skeletal muscle engineering, but the establishment of new fabrication methods that can meet the essential fabrication requirements for skeletal muscle bioengineering while recognizing scalability should also be striven for. Perhaps a viable new method for generating large-scale constructs with hypoxia-free cores would be to produce an acellular sacrificial block (either using 3D printing or molding technologies) with an array of dimensionally pre-defined cylindrical cavities where the cell-landed hydrogel can be dispensed and subsequently crosslinked prior to dissolving away the sacrificial support. This strategy not only would overcome the drawbacks of 3D bioprinting such as high cost and cell viability issues, but would also ensure the generation of large-scale 3D constructs with high structural stability in significantly reduced times that may not exceed the 1-h timeframe, thus overcoming the issue of hypoxia generation at the construct’s core during the fabrication process.

### Hypoxia generation during culture and potential solutions

Besides the potential risk of inducing a hypoxic core during fabrication, hypoxia may also be generated during culture. To prevent such an event from taking place, the hydrogel-based fabrication strategy should be able to pre-establish a well-defined pore volume within the resultant construct to allow for oxygen and nutrient diffusion at the deepest layers of the construct. Also, given that almost all hydrogels undergo a rapid cell-mediated contraction during culture [247–250], the fabrication strategy should be coupled with techniques that ensure maintaining the structural integrity of the pre-established pore volume within the resultant constructs to ensure constant diffusion of oxygen and nutrients. So far, hydrogel tethering is the most efficient technique to preserve the structural integrity of hydrogel-based constructs throughout the entire culture time because the constructs are being held tethered, which ensures that the hydrogel’s cell-mediated contraction is continuously being restricted [167]. Therefore, it would be highly beneficial if the hydrogel-based fabrication strategy is designed such that the resultant constructs are adaptable to this technique (hydrogel tethering).

### Hypoxia generation after transplantation and potential solutions

Upon transplantation, the construct is often packed within the defect area, which may deteriorate the existing pore volume, thus limiting the diffusion of oxygen to the construct’s core, especially in large muscle implants. One potential method to overcome the transport limitations is to pre-vascularize the construct to facilitate immediate oxygen transport upon implantation into the host. This can be achieved by incorporating endothelial cells during the fabrication process [32]. While this approach has improved graft survival and stability and enhanced the overall therapeutic effects [174], the inclusion of a secondary cell phenotype during the fabrication process is often complicated and time-consuming. Perhaps incorporating angiogenic growth factors [e.g., vascular endothelial growth factor (VEGF)] into the construct’s design may offer a simpler yet reliable approach to promote immediate angiogenesis within the transplanted construct. Alternatively, angiogenesis can be encouraged by utilizing a hydrogel biomaterial that can support angiogenesis. An ideal example would be ECM-derived hydrogels. The natural ECM is known to act as a reservoir for various growth factors, including VEGF [136]. In addition, previous literature has shown that ECM-derived hydrogels can still retain the growth factor content post-production [251]. However, there is no evidence that these approaches have been explored in the VML environment with large-scale skeletal muscle constructs (SMCs). Therefore, future studies should concentrate on investigating the potential of these methods to promote robust vascular regeneration following VML and to prevent the development of a hypoxic core in large-scale SMCs.

Beyond vascularization, the host immune response is a decisive determinant of the clinical success of hydrogel-based SMCs following implantation. Any implanted construct provokes a foreign-body response in which adsorbed proteins recruit monocytes that mature into macrophages and may fuse into foreign-body giant cells, potentially culminating in chronic inflammation and fibrous encapsulation that isolate the construct and impede its integration with host tissue [252]. Importantly, however, the immune response is not merely an obstacle to be suppressed; when appropriately directed, it is an active driver of muscle regeneration. In a traumatic VML model, biomaterial scaffolds were shown to establish a pro-regenerative immune microenvironment governed by a T helper 2 (Th2) pathway and interleukin-4–dependent polarization of macrophages toward a pro-healing (M2) phenotype, which proved critical for functional muscle recovery [253]. These findings underscore that the composition, degradation profile and biochemical presentation of the hydrogel should be engineered not only to minimize adverse chronic inflammation but also to steer macrophage polarization toward a constructive, regenerative response. In this regard, extracellular matrix (ECM)-derived hydrogels are particularly compelling, as their native compositional cues can favor a constructive remodeling response; a more in-depth treatment of the immunomodulatory behavior and host–material interactions of ECM-derived hydrogels in SMRE is provided in our previous review [136]. Because immunogenicity is further modulated by the chosen cell source—an aspect examined for each candidate population in Section 3 and summarized in Table 1—the co-design of an appropriately matched cell source and an immunomodulatory hydrogel represents an important and still under-explored priority for advancing large-scale SMCs toward clinical translation.

Another crucial consideration for the scalability of hydrogel-based SMCs is the selection of the appropriate cell source, given that hydrogel-based SMCs necessitate a relatively larger initial cell number compared to polymeric-based SMCs. Skeletal muscle precursors such as SSCs are by far the most potent cell source for SMRE. However, the necessity for invasive procedures to isolate them, low yield post-isolation and poor proliferative capacity *in vitro* significantly hamper their clinical and scalability potential. iPSCs, on the other hand, represent a viable alternative that has the potential to overcome the limitations associated with SSCs. Their unlimited self-renewal capacity, ability to proliferate indefinitely *in vitro* without losing their pluripotency, and robust myogenic differentiation potential render them highly suitable for clinical indications [254]. In addition, protocols for the mass expansion of iPSCs currently exist [255, 256], further facilitating their clinical utility and proving their scalability potential. Nevertheless, according to the current review, most SMRE-based studies predominantly concentrate on the utilization of SSCs, with less emphasis on iPSCs. Hence, future studies should contemplate incorporating iPSCs into the design of the constructs to enhance their overall scalability potential. Additionally, further investigation into the safety and efficacy of iPSCs is recommended before progressing to clinical translation.

Collectively, despite significant advancements in hydrogel-based skeletal muscle research, further investigation is undoubtedly necessary before these efforts can transition to clinical application. Given the current breakthroughs in research, it is anticipated that a viable strategy capable of fostering efficient skeletal muscle regeneration will emerge in the near future.
